# Chronic hypoxia leads to cognitive impairment by promoting HIF-2α-mediated ceramide catabolism and alpha-synuclein hyperphosphorylation

**DOI:** 10.1038/s41420-022-01260-6

**Published:** 2022-11-30

**Authors:** Gaifen Li, Jia Liu, Mengyuan Guo, Yakun Gu, Yuying Guan, Qianqian Shao, Wei Ma, Xunming Ji

**Affiliations:** 1 Beijing Institute of Brain Disorders, Laboratory of Brain Disorders, Ministry of Science and Technology, Collaborative Innovation Center for Brain Disorders, Beijing Advanced Innovation Center for Big Data-based Precision Medicine, Capital Medical University, Beijing, China; 2 Department of Neurosurgery, Xuanwu Hospital, Capital Medical University, Beijing, China

**Keywords:** Dementia, Pathogenesis, Stress and resilience, Molecular neuroscience

## Abstract

Chronic hypoxia leads to irreversible cognitive impairment, primarily due to hippocampal neurodegeneration, for which the underlying mechanism remains poorly understood. We administered hypoxia (13%) to C57BL mice for 1–14 days in this study. Chronic hypoxia for 7 or 14 d, but not 1 or 3 d, resulted in alpha-synuclein hyperphosphorylation at serine129 (α-Syn p-S129) and protein aggregation, hippocampal neurodegeneration, and cognitive deficits, whereas the latter could be prevented by alpha-synuclein knockdown or an administered short peptide competing at α-Syn S129. These results suggest that α-Syn p-S129 mediates hippocampal degeneration and cognitive impairment following chronic hypoxia. Furthermore, we found that chronic hypoxia enhanced ceramide catabolism by inducing hypoxia-inducible factor (HIF)-2α and HIF-2α-dependent transcriptional activation of alkaline ceramidase 2 (Acer2). Thus, the enzymatic activity of protein phosphatase 2A (PP2A), a specific phosphatase for α-syn, is inhibited, leading to the sustained induction of α-Syn p-S129. Finally, we found that intermittent hypoxic preconditioning protected against subsequent chronic hypoxia-induced hippocampal neurodegeneration and cognitive impairment by preventing α-Syn p-S129. These results proved the critical role of α-syn pathology in chronic hypoxia-afforded cognitive impairment and revealed a novel mechanism underlying α-syn hyperphosphorylation during chronic hypoxia. The findings bear implications in developing novel therapeutic interventions for chronic hypoxia-related brain disorders.

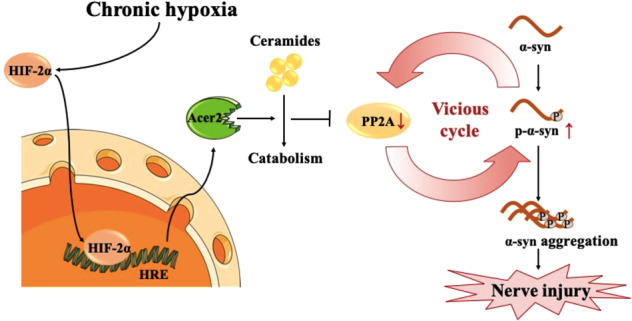

## Introduction

Hypoxia is an important stressor that induces cell damage, especially in the central nervous system (CNS), which is highly susceptible [1]. Neurons are the most abundant cell population in the CNS and are highly susceptible to hypoxia. Their damage induces the occurrence and development of various CNS diseases [2]. Chronic hypoxia, such as environmental hypoxia and tissue hypoxia, is common [3, 4]. Both environmental and tissue hypoxia cause neuronal damage. For example, environmental hypoxia caused by altitude exposure can cause irreversible impairment of cognitive ability or memory ability [5]. With aging, the brain’s ability to use oxygen gradually decreases, which leads to the development of a range of neurological diseases, such as neurodegenerative diseases [6]. However, the molecular mechanism of neuronal injury induced by hypoxia remains unclear, limiting the development of interventions for many neurological diseases.

The α-synuclein (α-syn) is a protein that is highly expressed in neurons. Although it has a powerful physiological function, more studies have focused on its pathological role, mainly because it is involved in the pathogenesis of several neurodegenerative diseases, such as Parkinson’s disease (PD) and dementia of Lewy body (DLB) [7, 8]. Abnormal changes in posttranslational modifications, especially phosphorylation at serine 129 (ser129) (p-α-syn), are critical for the transition of α-syn from a physiological to pathological function [9]. The proportion of p-α-syn in the normal state is very low, and an abnormal increase in p-α-syn induces the formation of pathological aggregates that contribute to neurotoxicity [10]. Previous studies have shown that hypoxia could promote α-syn phosphorylation and pathological aggregation [11], but its molecular mechanism is still unclear. Further exploration of this mechanism will be of great significance for understanding or intervening in hypoxia-related diseases and α-syn-related diseases.

In this study, we found that chronic hypoxia-induced irreversible cognitive impairment in mice by promoting α-syn phosphorylation at ser129 and abnormal aggregate formation in the hippocampus. Both α-syn knockout mice and mice administered a short peptide competing at α-syn ser129 were able to resist chronic hypoxia-induced cognitive impairment. Such results suggested that α-syn phosphorylation played an important role in chronic hypoxia-induced cognitive impairment. Our further study showed that chronic hypoxia-induced α-syn pathology promoted the hydrolytic metabolism of ceramide and decreased protein phosphatase 2A activity through activation of the hypoxia-inducible factor (HIF)-2α/alkaline ceramidase (Acer)2 signaling pathway. In conclusion, our study revealed a novel mechanism of neuronal injury induced by chronic hypoxia, highlighted the role of α-syn phosphorylation in this process, and provided a potential new target for the intervention of chronic hypoxia injury.

## Results

### Chronic hypoxia causes cognitive impairment in mice by promoting hippocampal neurodegeneration

**Fig. 1:**
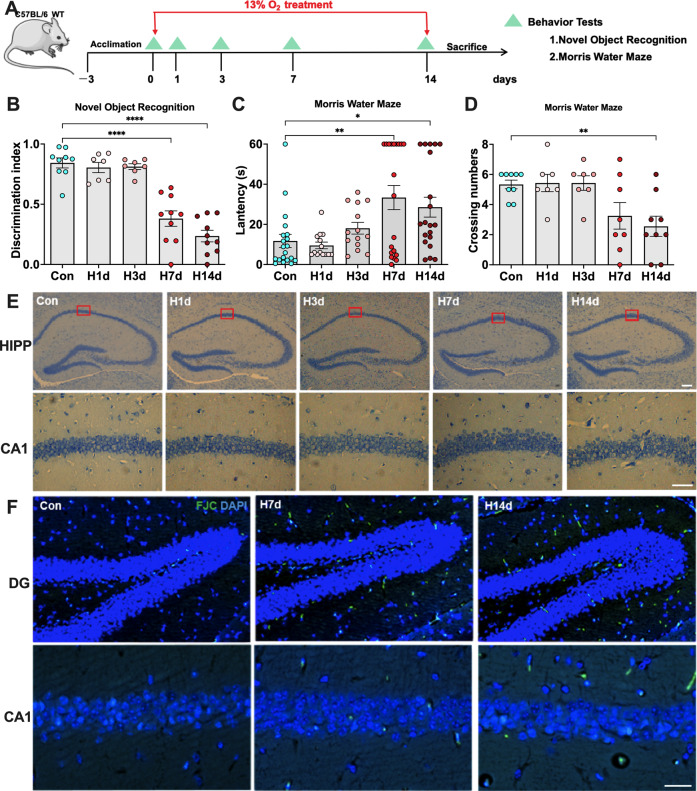
Chronic hypoxia causes cognitive impairment in mice by promoting hippocampal neurodegeneration. **A** Mice received continuous hypoxia-inducing treatment for 0, 1, 3, 7, and 14 days (Con, H1d, H3d, H7d, and H14d), and underwent behavioral tests at different time points. **B** Detection and statistical analysis of the novel object recognition tests in mice of each group. **C**, **D** Detection and statistical analysis of the Morris water maze tests in mice of each group. **E** Nissl staining was used to evaluate the arrangement of neurons in the hippocampal (HIPP) CA1 region of each group. **F** Fluoro-Jade C (FJC) staining was used to evaluate the neurodegeneration of the DG and CA1 regions in the hippocampus of each group. Data are expressed as the mean ± SEM (one-way ANOVA). *P < 0.05, **P < 0.01, ****P < 0.0001, *n* = 10. Bar = 50 μm in **E**, **F**.

Adult C57BL mice were continuously treated with 13% O_2_ for 0–14 days to construct a mouse hypoxia model. This hypoxic concentration is approximately equal to that at Lhasa on the Tibetan Plateau, the highest plateau city [12]. Mice at 0, 1, 3, 7, and 14 days of hypoxia (Con, H1d, H3d, H7d, H14d) underwent behavioral tests and postmortem histological tests to assess the neurological damage (Fig. 1A). New object recognition tests and the Morris water maze tests were used to assess the novel object exploration and spatial memory abilities. The results of these two behavioral tests showed that H1d and H3d mice did not experience cognitive decline, while H7d and H14d mice showed a significant weakening in novel object recognition and impaired spatial memory abilities (Fig. 1B–D). Mouse hippocampal neurons stained by Nissl staining showed that the hippocampal neurons of mice in the Con, H1d, and H3d groups had an orderly arrangement but a disordered arrangement after prolonged hypoxia, especially in the H14d group. These results are consistent with the cognitive decline observed in mice with chronic hypoxia (Fig. 1E). Fluoro-Jade C (FJC) staining showed neurodegeneration in the hippocampal CA1 and DG regions in mice in the H7d and H14d groups compared with the Con group (Fig. 1F). Therefore, these results suggested that chronic and persistent hypoxia-induced cognitive impairment in mice by inducing degeneration of hippocampal neurons.

### Chronic hypoxia promotes α-syn phosphorylation at ser129 and abnormal aggregation in the mouse hippocampus

**Fig. 2:**
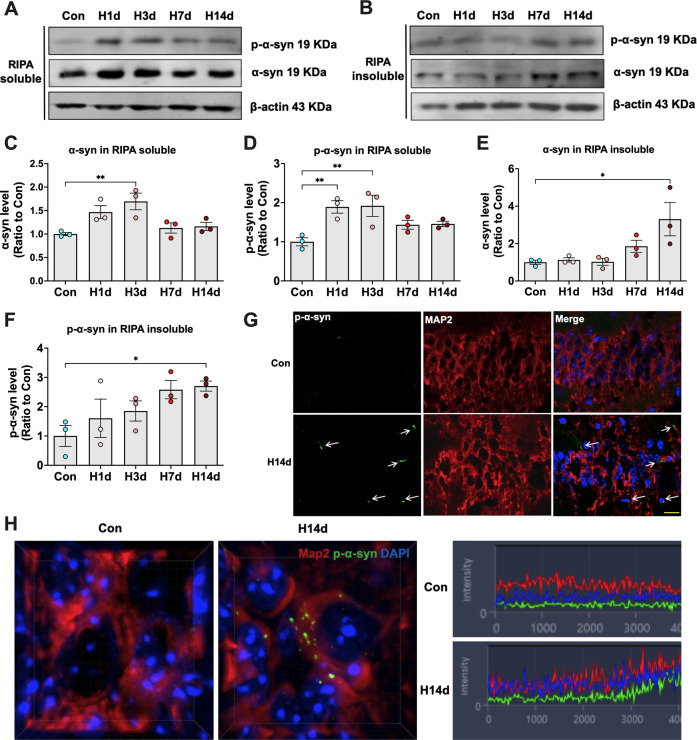
Chronic hypoxia promotes α-syn phosphorylation at ser129 and abnormal aggregation in the mouse hippocampus. **A**–**F** The levels of p-α-syn and α-syn in the RIPA-soluble and RIPA-insoluble fractions (8 M urea dissolved) from the hippocampi of mice in each group (Con, H1d, H3d, H7d, H14d) were detected by western blots with β-actin as the internal reference; Statistical analysis was performed. **G** The levels of p-α-syn and the neuronal marker Map2 in the hippocampus of the H14d group and Con group were detected by immunofluorescence. **H** The high-power three-dimensional imaging of p-α-syn immunofluorescence and the fluorescence intensity of each channel. Data are expressed as the mean ± SEM (one-way ANOVA). *P < 0.05, **P < 0.01, *n* = 3. Bar = 50 μm in **G**.

Hyperphosphorylation and abnormal aggregation of α-syn are closely related to neuronal degeneration. To investigate whether abnormal changes in α-syn were involved in neuronal injury induced by chronic hypoxia, we detected the levels of α-syn and p-α-syn in the hippocampi of mice in each group. The α-syn and p-α-syn levels in the Radio Immunoprecipitation Assay (RIPA)-soluble fraction from the hippocampi of mice increased significantly with hypoxic treatment for one day compared with the Con group (Fig. 2A, C, D). In addition, α-syn and p-α-syn levels increased in the insoluble fraction of RIPA following hypoxic treatment for 7 days, suggesting that hypoxia promoted the phosphorylation of α-syn and long-term hypoxia promoted α-syn to form abnormal aggregates in the insoluble fraction (Fig. 2B, E, F). Immunofluorescence staining was carried out to further observe whether the increased p-α-syn induced by hypoxia was aggregated. The results showed almost no significant p-α-syn staining in the hippocampal tissue of mice in the Con group. However, a large amount of p-α-syn staining aggregated in the hippocampal tissue of mice in the H14d group (Fig. 2G–H).

Primary neurons from mouse hippocampi were cultured for 7 days and then treated with 1% O_2_ for 0–6 h (Con, H1h, H2h, H4h, H6h). We tested cell viability and cytotoxicity of the neurons in each group by 3-(4,5)-dimethylthiahiazo (-z-y1)-3,5-di-phenytetrazoliumromide (MTT) and lactate dehydrogenase (LDH) assays. The results showed no significant decrease in cell viability within 2 h of hypoxic treatment but decreased cell viability after 4 h of hypoxia. We chose 4 h of hypoxia for subsequent experimental detection (Supplemental Fig. 1A, B). We used Propidium Iodide (PI)/Hoechst double staining to detect cell mortality. The results also showed that hypoxia for 4 h significantly increased the mortality of the primary neurons (Supplemental Fig. 1C, D). Similar to in vivo experiments, we also found that hypoxia promoted α-syn phosphorylation and increased its level in primary neurons (Supplemental Fig. 2). Immunofluorescence staining of α-syn and p-α-syn showed that aggregates formed in the cytoplasm and axons of neurons after hypoxic treatment. In particular, when p-α-syn formed aggregates, the neuron marker Map2 disappeared, indicating that the neurons were dead (Supplemental Fig. 3). These results suggested that chronic hypoxia resulted in an increase in p-α-syn phosphorylation and abnormal aggregation.

### The α-syn knockout mice or mice administered a competing peptide targeting ser129 resist chronic hypoxia-induced cognitive impairment

**Fig. 3:**
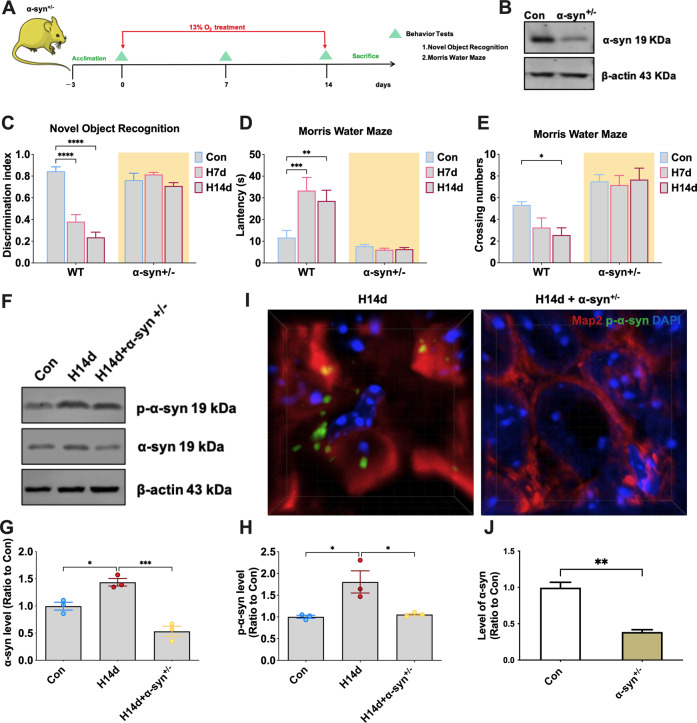
Mice with α-syn knockout resist chronic hypoxia-induced cognitive impairment. **A**, **B**, **J** Heterozygous mice with α-syn knockout received continuous hypoxia-inducing treatment for 0, 7, and 14 days (Con, H7d, and H14d), and then underwent behavioral tests at different time points. **C** Detection and statistical analysis of the novel object recognition tests in mice of each group. **D**, **E** Detection and statistical analysis of the Morris water maze tests in mice of each group. **F**–**H** The levels of p-α-syn and α-syn in the hippocampi of mice in the Con, H14d, and H14d+α-syn^+/-^ groups were detected by western blots with β-actin as the internal reference; Statistical analysis was performed. **I** The high-power three-dimensional imaging of p-α-syn immunofluorescence. In **C**–**E**, data are expressed as the mean ± SEM (two-way ANOVA), *P < 0.05, **P < 0.01, ***P < 0.001, ****P < 0.0001, *n* = 10. In **G**–**H**, data are expressed as the mean ± SEM (one-way ANOVA), *P < 0.05, ***P < 0.001, *n* = 3. In **J**, data are expressed as the mean ± SEM (unpaired *t*-test), **P < 0.01, *n* = 3.

The above studies showed that chronic hypoxia-induced cognitive impairment in mice and was accompanied by abnormal changes in α-syn. To investigate whether abnormal changes in α-syn were a critical factor in chronic hypoxia-induced nerve injury, we used α-syn knockout mice. Heterozygous α-syn knockout mice received the same chronic hypoxia-inducing treatment. New object recognition tests and Morris water maze tests were used to detect the cognitive behavior of the mice (Fig. 3A). The α-syn levels in the hippocampi of heterozygous α-syn knockout mice were significantly lower than in wild-type mice (Fig. 3B, J). The behavioral test results showed that α-syn knockdown significantly alleviated the chronic hypoxia-induced decline in exploration ability to novel objects and spatial memory impairment (Fig. 3C–E). These results suggested that α-syn played a critical role in neuronal injury induced by chronic hypoxia. western blot analysis also showed that chronic hypoxia did not induce an excessive increase in α-syn and p-α-syn in the hippocampi of heterozygous α-syn knockout mice compared with wild-type mice (Fig. 3F–H). Immunofluorescence staining also showed that chronic hypoxic treatment in heterozygous α-syn knockout mice did not result in significant p-α-syn aggregate formation (Fig. 3I). Similarly, in primary neurons, we also found that knockdown of A-SYN by siRNA lentivirus mitigated hypoxia-induced neuronal death (Supplemental Fig. 4). These data suggest that abnormal alterations in α-syn are important in chronic hypoxia-induced nerve injury.

**Fig. 4:**
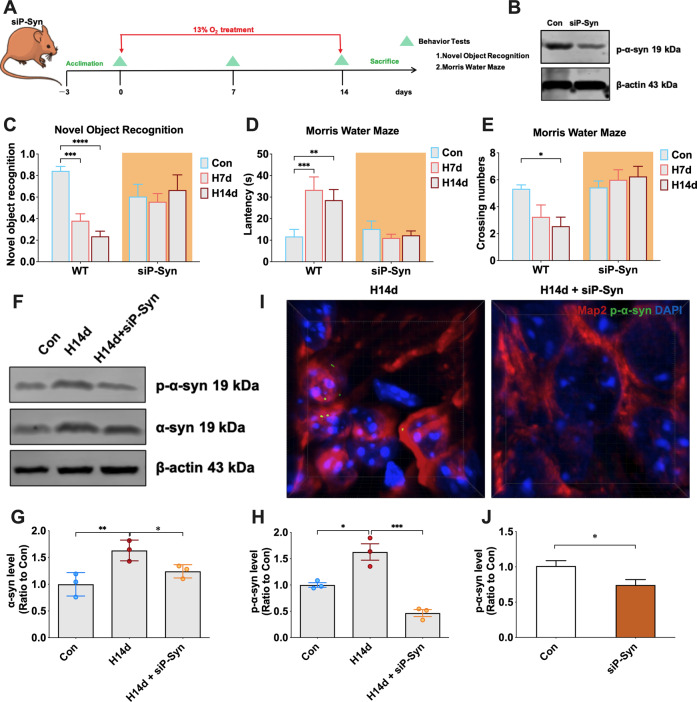
Mice administered with competing peptides targeting ser129 resist chronic hypoxia-induced cognitive impairment. **A**, **B**, **J** Mice were administered competing peptides targeting ser129 and then received continuous hypoxia-inducing treatment for 0, 7, and 14 days (Con, H7d, and H14d) before undergoing behavioral tests at different time points. **C** Detection and statistical analysis of the novel object recognition tests in mice of each group. **D**, **E** Detection and statistical analysis of the Morris water maze tests in mice of each group. **F**–**H** The levels of p-α-syn and α-syn in the hippocampi of mice in the Con, H14d, and H14d+siP-Syn groups were detected by western blots with β-actin as the internal reference; statistical analysis was performed. **I** The high-power three-dimensional imaging of p-α-syn immunofluorescence. In **C**–**E**, data are expressed as the mean ± SEM (two-way ANOVA), *P < 0.05, **P < 0.01, ***P < 0.001, ****P < 0.0001, *n* = 10. In **G**–**H**, data are expressed as the mean ± SEM (one-way ANOVA), *P < 0.05, **P < 0.01, ***P < 0.001, *n* = 3. In **J**, data are expressed as the mean ± SEM (unpaired *t*-test), *P < 0.05, *n* = 3.

Hyperphosphorylation of α-syn at ser129 is an important step in the transformation of α-syn both from soluble monomers to insoluble aggregates and from a non-toxic to a toxic form. Our previous experiments have confirmed that chronic hypoxia promotes increased levels of α-syn phosphorylation. Therefore, we further explored whether preventing α-syn phosphorylation would resist chronic hypoxia-induced nerve damage. We used a previously reported small-molecule peptide that competently inhibits α-syn hyperphosphorylation at ser129 [13] and administered it to mice via the tail vein (Fig. 4A). Western blots showed that p-α-syn levels were significantly reduced in the peptide-treated mice (siP-Syn) (Fig. 4B, J). The siP-Syn mice were also subjected to chronic hypoxia as in the previous experiments. The behavioral tests showed that, unlike wild-type mice, siP-Syn mice were able to resist cognitive impairment induced by chronic hypoxia (Fig. 4C–E). This occurred mainly due to the resistance of siP-Syn mice to the hyperphosphorylation and abnormal aggregation of α-syn in the hippocampus induced by chronic hypoxia (Fig. 4F–I). These results suggested that chronic hypoxia-induced abnormal aggregation of α-syn by promoting its hyperphosphorylation, which was a key factor in inducing nerve damage and cognitive impairment in mice.

### Chronic hypoxia induces a vicious cycle of decreased PP2A activity and increased p-α-syn

**Fig. 5:**
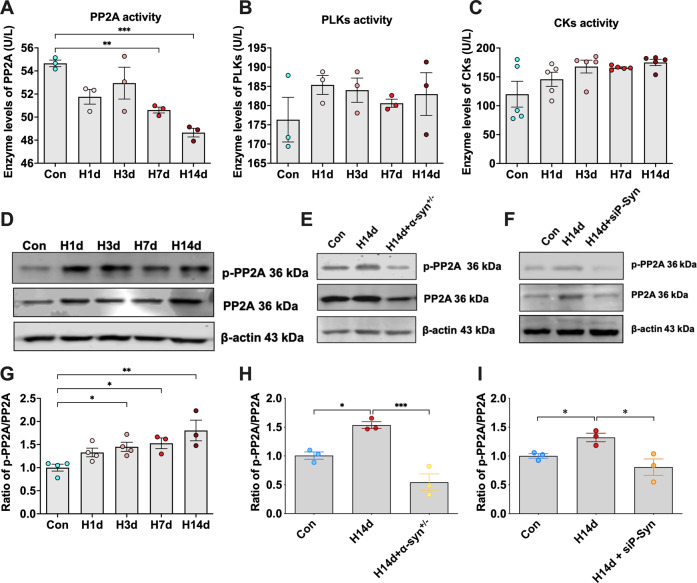
Chronic hypoxia induces a vicious cycle of decreased PP2A activity and increased p-α-syn. **A**–**C** Enzyme activities of PP2A, PLK, and CK in the hippocampi of mice in each group (Con, H1d, H3d, H7d, H14d) were detected and statistically analyzed. **D**, **G** The levels of p-PP2A and PP2A in the hippocampi of mice of different hypoxic time groups (Con, H1d, H3d, H7d, H14d) were detected by western blots with β-actin as the internal reference; Statistical analysis was performed. **E**, **H** The levels of p-PP2A and PP2A in the hippocampi of mice in the Con, H14d, and H14d+α-syn^+/−^ groups were detected by western blots with β-actin as the internal reference; Statistical analysis was performed. **F**, **I** The levels of p-PP2A and PP2A in the hippocampi of mice in the Con, H14d, and H14d + H14d+siP-Syn groups were detected by western blots with β-actin as the internal reference; Statistical analysis was performed. Data are expressed as the mean ± SEM (one-way ANOVA), *P < 0.05, **P < 0.01, ***P < 0.001, *n* = 3–5.

We have found that hypoxia promoted α-syn phosphorylation, but the molecular mechanism remained unclear. Phosphorylation of α-syn is regulated by kinases and phosphatases. Polo-like kinase (PLK) and creatine kinase (CK) are the main kinases promoting α-syn phosphorylation [14], while PP2A is the main phosphatase promoting α-syn dephosphorylation [15]. To explore how hypoxia causes α-syn hyperphosphorylation, we tested the enzyme activities of these enzymes. The results showed that the activity of kinases CK and PLK in the hippocampal tissue of mice in the hypoxic group increased compared with the control group. However, there was no statistical significance. The activity of phosphatase PP2A in the hypoxia group decreased significantly (Fig. 5A–C). We further detected the level of PP2A and its phosphorylation at tyrosine 307 (p-PP2A) by western blotting, which characterized its inactivity. The results showed that the p-PP2A/PP2A ratio increased significantly after hypoxia, indicating that the PP2A activity decreased (Fig. 5D, G). Interestingly, we found that α-syn knockout mice or siP-Syn mice were resistant to a chronic hypoxia-induced decline in PP2A activity, showing no increase in the p-PP2A/PP2A ratio after hypoxia (Fig. 5E, F, H, I).

Similarly, in primary neurons, we also found that hypoxic treatment resulted in decreased activity in PP2A in neurons. We treated cells with both iHAP1 [16], a PP2A agonist, and LB-100 [17], an inhibitor of PP2A, and showed that iHAP1 mediated the activation of PP2A mitigated hypoxia-induced neuronal death (Supplemental Fig. 5).

These results suggested that chronic hypoxia promotes hyperphosphorylation of α-syn through inhibition of PP2A activity and further inhibition of PP2A activity through p-α-syn, creating a vicious cycle.

### Chronic hypoxia promotes ceramide hydrolytic metabolism by activating the HIF-2α/Acer2 signaling pathway, and thereby downregulates PP2A activity

**Fig. 6:**
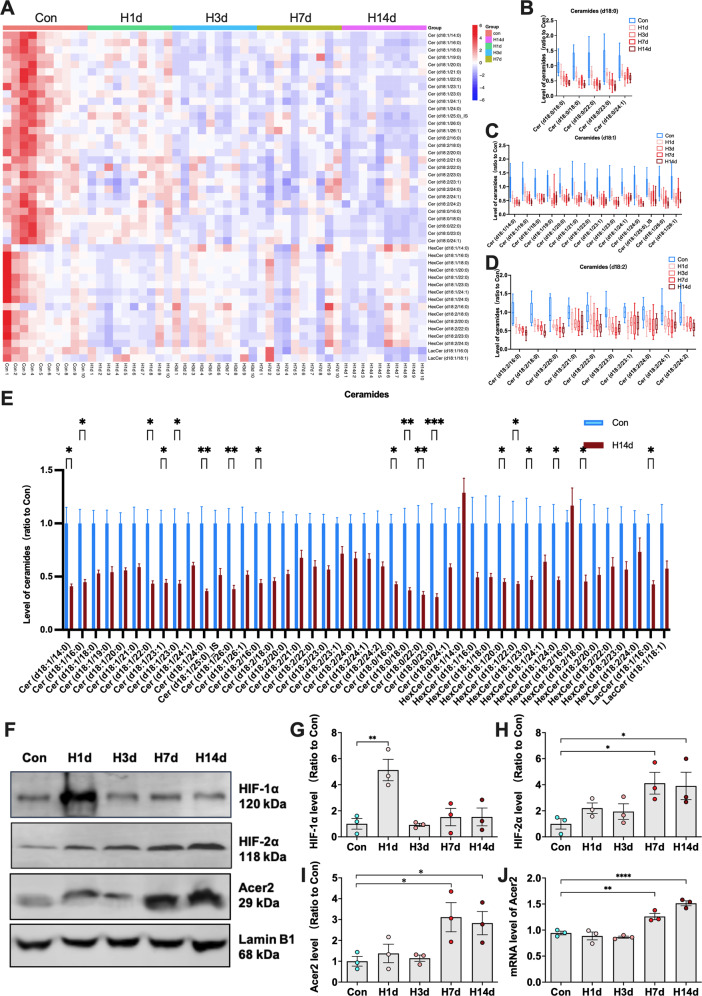
Chronic hypoxia promotes ceramide hydrolytic metabolism by activating the HIF-2α/Acer2 signaling pathway, thereby downregulating PP2A activity. **A**–**E** Metabolomics of ceramides in the hippocampal tissue of mice with hypoxia for different periods (Con, H1d, H3d, H7d, H14d); heat map and statistical analysis of different subtypes of ceramides in each group. **F**–**I** The levels of HIF-1α, HIF-2α, and Acer2 in the nuclei of the hippocampi of mice of different hypoxic time groups (Con, H1d, H3d, H7d, H14d) were detected by western blots with β-actin as the internal reference; Statistical analysis was performed. **J** The mRNA levels of Acer2 in the hippocampi in each group were detected by RT-PCR and statistically analyzed. In **B**–**E**, data are expressed as the mean ± SEM (two-way ANOVA), *P < 0.05, **P < 0.01, ***P < 0.001, *n* = 10. In **G**–**J**, data are expressed as the mean ± SEM (one-way ANOVA), *P < 0.05, **P < 0.01, ****P < 0.0001, *n* = 3.

The above study found that hypoxia caused α-syn hyperphosphorylation by inhibiting PP2A activity. Therefore, we further explored the underlying mechanism. Ceramides are essential for maintaining PP2A activity in vivo [18]. For this reason, we tested different species of ceramides by using targeted metabolomics. The results showed that hypoxia reduced the levels of almost all ceramide species, and this was significantly correlated with the duration of hypoxia (Fig. 6A–E). Recently, it was reported that the ceramide hydrolase alkaline ceramidase (Acer)2 is a novel downstream target molecule of hypoxia-inducible factor (HIF)-2α [19]. For this reason, we investigated whether hypoxia caused a reduction in ceramide levels through this pathway. HIF-1α, HIF-2α, and Acer2 levels were detected by western blots. The results showed that HIF-1α increased temporarily in the H1d group and then decreased in groups with longer hypoxia (Fig. 6F–G). In contrast, HIF-2α, which is more indicative of chronic hypoxia, increased steadily with prolonged hypoxia time (Fig. 6F, H). Similarly, Acer2, downstream of HIF-2α, also showed a sustained and stable increase after hypoxia at both protein and mRNA levels (Fig. 6F, I). To further confirm that the increased Acer2 level after hypoxia was caused by transcriptional activation, we detected the expression of Acer2 by RT-PCR and obtained similar results. That is, chronic hypoxia steadily promoted the expression of Acer2 (Fig. 6J). These results suggested that chronic hypoxia promoted the expression of Acer2 by activating HIF-2α. Increased Acer2 promoted the hydrolytic metabolism of ceramides. It may be an important reason for the inhibition of PP2A activity and the hyperphosphorylation of α-syn.

### Chronic hypoxia-induced α-syn pathology and cognitive impairment in mice remain irreversible after reoxygenation

**Fig. 7:**
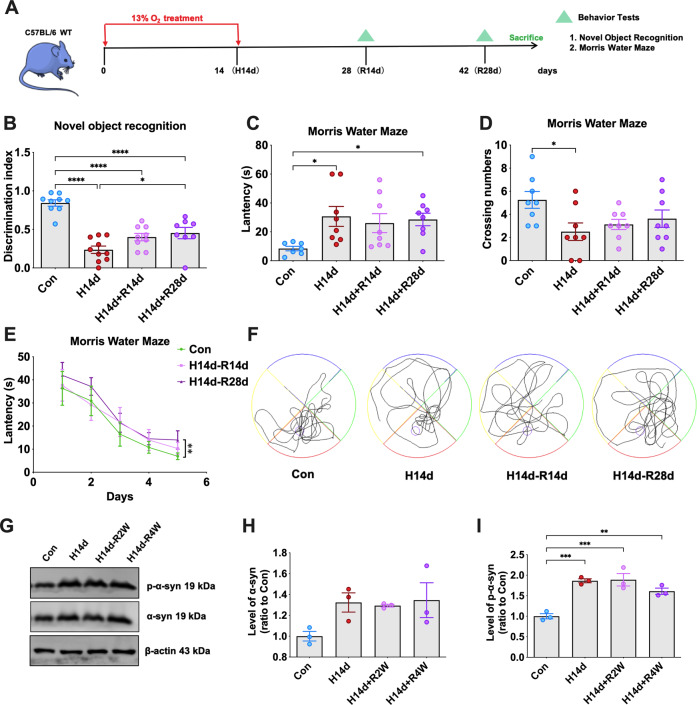
Chronic hypoxia-induced α-syn pathology and cognitive impairment in mice remain irreversible after reoxygenation. **A** Mice received continuous hypoxia-inducing treatment for 14 days (H14d), reoxygenation for 14 days (H14d + R14d) or 28 days (H14d + R28d), and then underwent behavioral tests at different time points. **B** Detection and statistical analysis of the novel object recognition tests in mice of each group. **C**–**F** Detection and statistical analysis of the Morris water maze tests in mice of each group. **G**–**I** The levels of p-α-syn and α-syn in the hippocampi of mice in the Con, H14d, H14d + R2W, and H14d + R4W groups were detected by western blots with β-actin as the internal reference; statistical analysis was performed. Data are expressed as the mean ± SEM (one-way ANOVA), *P < 0.05, **P < 0.01, ***P < 0.001, ****P < 0.0001, *n* = 10 in **B**–**E**, *n* = 3 in **G**–**I**.

We have found that chronic hypoxia promoted the abnormal pathology of α-syn through an HIF-2α-dependent pathway, which is an important link in inducing neuronal injury and cognitive impairment in mice. However, it is not known whether α-syn pathology and cognitive impairment induced by chronic hypoxia are reversible or irreversible. Therefore, the hypoxic mice were reoxygenated for a long time to observe the recovery of the mice (Fig. 7A). The results showed that the mice that experienced hypoxia for 14 days still had obvious cognitive-behavioral impairment after reoxygenation for 28 days, suggesting that the neurological damage induced by chronic hypoxia was irreversible (Fig. 7B–F). By western blot analysis, we still found a large amount of p-α-syn in the hippocampal tissues of reoxygenated mice, suggesting that α-syn pathology induced by chronic hypoxia was also irreversible for an extended period of time (Fig. 7G–I). These results suggested that α-syn pathology and cognitive impairment induced by chronic hypoxia were irreversible over an extended reoxygenation period in mice.

### Intermittent hypoxia preconditioning alleviates chronic hypoxia-induced cognitive impairment in mice by inhibiting α-syn phosphorylation and aggregation

**Fig. 8:**
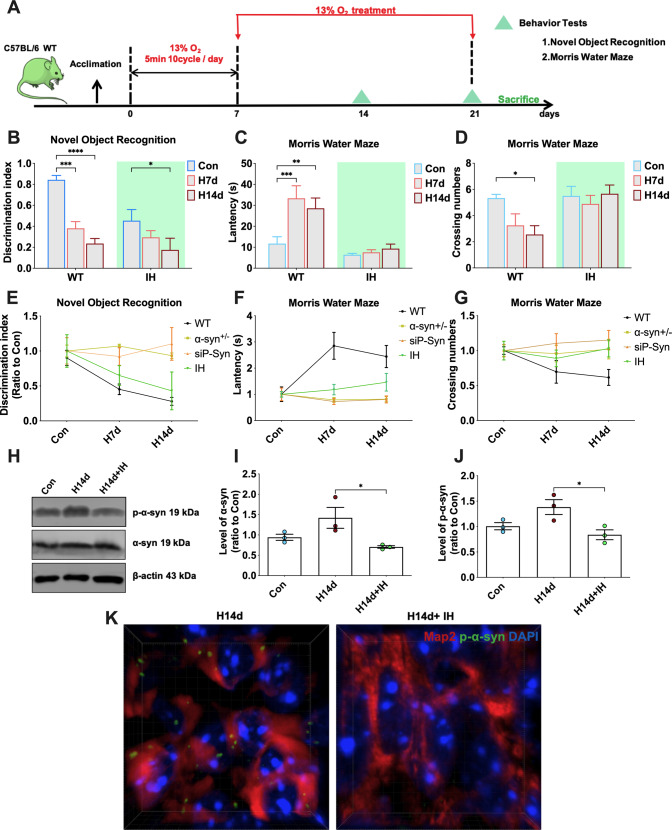
Intermittent hypoxia preconditioning (IHPC) alleviates chronic hypoxia-induced cognitive impairment in mice by inhibiting α-syn phosphorylation and aggregation. **A** Mice were pretreated with intermittent hypoxia (IH) for 7 days by 10 cycles of intermittent hypoxia per day, each of which was 13% O_2_ for 5 min followed by 21% O_2_ for 5 min. After pretreatment, the mice were treated with continuous hypoxic conditions for 7 days and 14 days, and underwent behavioral tests at different time points. **B** Detection and statistical analysis of the novel object recognition tests in mice of each group. **C**, **D** Detection and statistical analysis of the Morris water maze tests in mice of each group. **E**–**G** The differences in mice with α-syn knockout, administered with a competing peptide targeting ser129, and preconditioned with intermittent hypoxia against persistent hypoxic-induced cognitive impairment were compared. **H**–**J** The levels of p-α-syn and α-syn in the hippocampi of mice in the Con, H14d, and H14d+IH groups were detected by western blots with β-actin as the internal reference; statistical analysis was performed. **K** The high-power three-dimensional imaging of p-α-syn immunofluorescence. Data are expressed as the mean ± SEM (one-way ANOVA), *P < 0.05, **P < 0.01, ***P < 0.001, ****P < 0.0001, *n* = 10 in **B**–**G**, *n* = 3 in **H**–**K**.

The above study found that chronic persistent hypoxia-induced irreversible cognitive impairment in mice. Compared with persistent hypoxia, intermittent hypoxia may be a more moderate pattern of hypoxia. Previous studies have proposed that intermittent hypoxia preconditioning (IHPC) could increase the body’s tolerance to subsequent more severe injury [20]. Therefore, we investigated whether IHPC could resist the α-syn pathology and cognitive impairment in mice induced by chronic persistent hypoxia. Our IHPC treatment pattern was as follows: mice were treated with 10 cycles of intermittent hypoxia per day. Each cycle of 13% O_2_ hypoxic treatment for 5 min was followed by 21% O_2_ normoxia for 5 min. Mice were treated with IHPC for a total of 14 days, followed by continuous treatment for another 14 days in 13% O_2_ hypoxic conditions (Fig. 8A). Novel object recognition tests and the Morris water maze tests showed that IHPC significantly alleviated cognitive impairment induced by persistent hypoxia in mice (Fig. 8B–G). We detected α-syn and p-α-syn in the hippocampal tissues of mice by western blots and immunofluorescence staining. The results showed that IHPC could significantly alleviated α-syn pathology induced by chronic hypoxia (Fig. 8H–K). These results suggested that IHPC alleviated chronic hypoxia-induced cognitive impairment and α-syn pathology in mice.

## Discussion

In this study, we found that chronic hypoxia-induced irreversible cognitive impairment in mice with α-syn phosphorylation and aggregation playing a crucial role. Mechanically, chronic hypoxia promoted the transcriptional expression of Acer2 by activating HIF-2α. Increased Acer2 resulted in the hydrolytic metabolism of ceramide and subsequent decreased activity of PP2A. These changes induced α-syn phosphorylation and abnormal aggregation, resulting in neurological damage. These findings revealed a novel mechanism of cognitive impairment induced by chronic hypoxia. In addition, we found that IHPC significantly alleviated chronic hypoxia-induced α-syn pathology and cognitive impairment in mice, which provided a novel perspective for the intervention of hypoxia-related diseases.

Chronic hypoxia is common in life and includes environmental hypoxia, such as altitude exposure, and hypoxia of the body or organs during aging and disease [21]. The brain is very sensitive to hypoxic stress. Both environmental hypoxia and body hypoxia cause neuronal damage in the brain [22]. However, the mechanism remains unclear. Our study first highlighted the importance of α-syn phosphorylation and aggregation in chronic hypoxia-induced cognitive impairment, showing that both α-syn knockout and p-α-syn competing interference resisted chronic hypoxia-induced neuronal injury. Furthermore, we systematically revealed the molecular mechanism of chronic hypoxia-induced α-syn pathology and neuronal injury. Compared with the acute hypoxic response mediated mainly by HIF-1α, HIF-2α is involved mainly in the chronic hypoxic response [23]. Our study found that chronic hypoxia promoted HIF-2α rather than HIF-1α entry into the nucleus, and nucleation of HIF-2α resulted in transcriptional expression of Acer2, a newly discovered downstream target of HIF that was reported in adipocytes [19, 24]. As an important hydrolytic enzyme of ceramides [25], increased Acer2 promotes the hydrolytic metabolism of ceramides, which is an important reason for the significantly decreased ceramide levels observed after hypoxic treatment. The decrease in ceramides, as the main maintainer of PP2A activity in the brain [18], resulted in the inhibition of PP2A activity. PP2A is the only phosphatase responsible for α-syn dephosphorylation [15]. Therefore, the decreased activity of PP2A induced by chronic hypoxia is an important factor for α-syn hyperphosphorylation and abnormal aggregation. Interestingly, we also found that the hypoxia-induced decline in PP2A activity was reversed when α-syn or p-α-syn was interfered with, suggesting that hypoxia-induced PP2A inhibition and α-syn pathology are mutually reinforcing vicious cycles. This hypothesis has been confirmed by previous studies [26, 27]. In conclusion, our study highlighted the role of α-syn pathology in chronic hypoxia-induced cognitive impairment and revealed a novel underlying mechanism, providing reliable targets for future research.

The α-syn is a protein that is abundant in the brain and is pathologically involved in a variety of neurological diseases. PD and DLB are the most studied diseases of α-syn at present [28, 29]. Their typical pathological feature is the deposition of intracellular Lewy bodies, the main component of which is p-α-syn. A few previous studies have mentioned the relationship between hypoxia and α-syn. For example, hypoxia can increase the expression and aggregation of α-syn in HEK293 cells [11, 30]. There has also been a study showing increased plasma α-syn levels in patients with chronic hypoxia [31]. A recent review has suggested that improving hypoxia adaptation may be an important strategy for PD intervention [32]. In our study, we found that hypoxia significantly promoted the phosphorylation of α-syn at ser129, a form of α-syn that was strongly associated with the above diseases. These results suggested that hypoxia stress may play an initiating or promoting role in the pathogenesis of α-syn-related diseases such as PD and DLB. These findings provided a novel perspective for further research and interventions for these diseases.

Compared with persistent hypoxia, intermittent hypoxia is a moderating pattern of hypoxia, and many previous studies have found that IHPC can resist subsequent more severe ischemic hypoxic injury [33, 34]. IHPC has been reported to play protective roles in various neurological diseases [20], it is considered as a potential non-drug intervention [35]. The protective effects of intermittent hypoxia in ischemic stroke have been extensively studied, and IHPC has been shown to reduce the infarct size and improve neurological function in stroke models [36–38]. Several studies have shown that IHPC exerted a neuroprotective effect in models of multiple sclerosis by suppressing inflammation and protecting the blood-brain barrier [39]. IHPC treatment also reduced amyloid pathology and improved cognitive performance in AβPP/PS1 transgenic mice of Alzheimer’s disease [40]. In this study, we found that IHPC alleviated chronic hypoxia-induced cognitive impairment in mice and resisted α-syn pathology, which provided a potential direction for the intervention of both hypoxia-related diseases and α-syn-related diseases, such as Parkinson’s disease. It is important to note that the IHPC we used was a more moderate model having alternating hypoxia for 5 min and normoxia for 5 min over 10 cycles per day for 14 days. This did not cause additional damage. It is quite different from the intermittent hypoxic conditions associated with some diseases, such as obstructive sleep apnea syndrome.

In summary, our study highlighted the importance of α-syn pathology in chronic hypoxic injury, elucidated its molecular mechanisms, and provided a reliable molecular target for subsequent research. Meanwhile, we also revealed the role of IHPC in resistance to chronic hypoxic injury, which provided a new perspective for the intervention of many hypoxia-related diseases.

## Materials and methods

### Animals

Adult male C57BL mice were purchased from SPF Biotechnology (Beijing, China). α-Syn knockout mice were provided by Professor Yang Hui of Capital Medical University. All animals were housed at room temperature under a 12/12 h light/dark cycle and had free access to food and water. All animal experiments were approved by the Animal Care and Use Committee of the Institute of Animal Management, Capital Medical University (permit no. AEEI-2021-058), and conducted in accordance with ethical requirements and ARRIVE guidelines.

### Hypoxic treatment

All mice were randomly assigned to the control group and each model group. Hypoxic mice were administered hypoxic treatment in a closed hypoxic chamber (China Innovation Instrument Co., Ltd, Ningbo, Zhejiang, China), which accurately set the desired hypoxic concentration and pattern. For chronic hypoxia, mice were treated continuously with 13% O_2_ for 1, 3, 7, and 14 days. The hypoxic chamber was opened briefly for food and water additions every 3 days. Intermittent hypoxic mice were treated with 10 cycles of 5-min 13% O_2_ (hypoxia) and 5-min 21% O_2_ (normoxia) per day for 14 days, followed by an additional continuous hypoxic treatment. Hypoxic-reoxygenated mice were fed normoxic for 28 days after receiving chronic hypoxia.

### The siP-Syn peptide administration

The polypeptide we used was based on a previous study [13]. The sequence was 5-FITC-(Acp)-YGRKKRRQRRRYEMPSEEGYQD. The peptide was formed by fusing the HIV Tat (transcriptional counter-activator) signal (YGRKKRRQRRR) with a competing peptide spanning amino acids 125 to 135 (YEMPSEEGYQD), the phosphorylation site (ser129) in α-syn (siP-Syn).

The siP-Syn peptide (Chinapeptides, Shanghai, China) was injected via the tail vein every 3 days for 6 weeks at a dose of 10 mg/kg. The mice were treated in a hypoxia-inducing environment and tested for behavior two weeks after injection. Polypeptides were stored at −20 °C and protected from light. Repeated freezing and thawing were prevented.

### Behavioral tests

The cognitive function of the mice in each group was evaluated by novel object recognition tests and Morris water maze tests. For the novel object recognition tests, the experimental device was a rectangular box with a length, width, and height of 40 cm. It had no lid, and the camera was placed on the top. The experiment was divided into three stages: adaptation stage, familiarity stage, and experimental stage. Adaptation stage: Each mouse was put into the experimental device and left to explore freely for 5 min to adapt. Familiarity stage: Two identical objects A (old objects) were placed in the experimental device, and the mice were allowed to explore freely for 5 min. Experimental stage: One object A was removed from the experimental device, and a new object B was put into it (the color and shape were different from A). The mice were put into the experimental device and left to move freely for 5 min. The time that the mice explored the old and new objects were recorded. Discrimination index = (new object recognition time − object recognition time)/(new object recognition time + object recognition time). In behavioral tests, mice with cognitive impairment at a baseline were excluded. All behavioral videos were blindly analyzed by researchers other than the operator.

The water maze device was a circular device with a diameter of 120 cm. It was divided into four quadrants and had a small platform. Each quadrant had a different color and marker shape for spatial memory recognition by the mice. Water was added to the pool until it was 1–2 cm higher than the platform. The water temperature was about 25 °C. Training stage: The mice were placed in the pool at the same position in one of the four quadrants, and the time spent finding the platform was recorded. If the mice could not find the platform within 60 s, the time spent finding the platform was recorded as 60 s. The mice were then guided to swim to the platform and allowed to stay there for 5 s. The training was performed once a day for 5 days in total. Test stage: On day 6, the platform was removed and the mice were placed in the quadrant farthest from the platform. The time at which the mice first found the location of the original platform and the number of times of crossing within 60 s were recorded. In behavioral tests, mice with cognitive impairment at a baseline were excluded. All behavioral videos were blindly analyzed by researchers other than the operator.

### Nissl staining

Brain tissue from the mice were cut into 10 μm sections on a frozen slicer and pasted on a slide. The sections were then fixed in 70% ethanol and successively dehydrated in 100% ethanol, 90% ethanol, 80% ethanol, and 70% ethanol for 2 min each. After the xylene became transparent, the sections were placed in 1% tar purple (Solarbio, G1430) and incubated for 30 min. The sections were cleaned with distilled water and separated with 70% alcohol for several minutes. The sections were dehydrated with 70% ethanol, 80% ethanol, and 95% ethanol for 2 min each, dehydrated with 100% ethanol, and sealed with neutral gum.

### Separation of soluble and insoluble components

Single mouse hippocampal tissue was isolated, and 300 μl mixed protein lysis solution with RIPA (Applygen, C1053), a protease inhibitor (Merck, 539134), and a phosphatase inhibitor (Merck, 524628) (=100:1:2) were added. After ultrasonic crushing, ice cracking was performed for 30 min. The preparation was centrifuged at 4 °C at 12,000 rpm for 15 min. The supernatant contained the RIPA-soluble protein components. The precipitate at the bottom of the tube was the insoluble fraction. Urea (8 M) was added to the precipitate to dissolve the RIPA-insoluble protein components.

### Separation of nuclear components

Nuclear separation was performed using a nuclear separation kit (Applygen, P1202) according to the instructions. Briefly, the mouse hippocampus was put into a pre-cooled glass homogenizer, and 500 μL of Buffer1 reagent was added. After grinding and mixing, the homogenate was placed on ice for 10 min for full lysis. The homogenate was transferred to a new pre-labeled centrifuge tube and centrifuged at 800 × *g* for 5 min at 4 °C. After centrifugation, the nuclear crude extract was at the bottom of the tube. Then, 500 μL Buffer2 was added to the bottom of the tube containing the crude nuclear extract. The crude nuclear extract was suspended and mixed by shock. Following centrifugation at 4000 × *g* at 4 °C for 5 min, the supernatant was discarded. Then, 500 μL Buffer2 was added again after suspension and centrifuged again to obtain relatively pure nuclei. Finally, 50 μL suspension buffer was used to re-suspend and obtain complete and unbroken nuclei.

### Western blots

Mouse hippocampus protein lysates were resolved by sodium dodecyl sulfate-polyacrylamide gel electrophoresis (SDS-PAGE) and subsequently immunoblotted onto polyvinylidene difluoride (PVDF) membranes. Membranes were blocked with 5% nonfat milk at room temperature for 1 h. After TBST washing (three times, 5 min per wash), the membranes were incubated with the indicated primary antibodies at 4 °C overnight with shaking. The primary antibodies included β-actin (Sigma-Aldrich, A5316), lamin B1 (HUABIO Antibodies, ET1606-27), HIF-1α (Abcam, ab228649), HIF-2α (Novus, NB100-122), Acer2 (PA5-39016), PP2A (Invitrogen, MA5-32920), p-PP2A (Invitrogen, MA5-32158), α-syn (BD, 8249518), and p-α-syn (Abcam, ab51253). After incubation, the membranes were washed three times and then incubated at room temperature for 1 h with secondary antibodies, including IRDye 680RD goat anti-mouse IgG (H + L) (Licor, 926-68070), IRDye 680RD goat anti-rabbit IgG (H + L) (Licor, 926-68071), IRDye 800CW goat anti-mouse IgG (H + L) (Licor, 926-32210), IRDye 800CW goat anti-rabbit IgG (H + L) (Licor, 926-32211). Membranes were scanned using a detection system (Odyssey, USA), and band intensities were normalized to β-actin. Statistical analyses were performed using ImageJ and GraphPad software.

### Immunofluorescence

Mice brain sections were boiled in citric acid buffer (pH = 6.0) for 10 min for antigen repair. Sections were placed in 0.01 M PBS and washed three times for 10 min each. Sections were placed in 1% PBST and incubated at room temperature for 30 min. Sections were then placed in 5% BSA and sealed for 2 h at room temperature. The sections were then incubated overnight at 4 °C in primary antibody: p-α-syn (WAKO, 015-25191) or MAP2 (Abcam, ab32454). The sections were washed in 0.01 M PBS three times for 10 min each. Sections were placed in fluorescent secondary antibody and incubated at room temperature for 2 h, protected from light. The secondary antibodies were goat anti-mouse IgG (H + L) highly cross-adsorbed secondary antibody, Alexa Fluor 488 (Invitrogen, A11029), and goat anti-rabbit IgG (H + L) highly cross-adsorbed secondary antibody, Alexa Fluor 594 (Invitrogen, A11037). DAPI was used for nuclei staining before sections were mounted (Sigma, D9542). A fluorescence microscope was used to observe and photograph the images.

### Enzyme activity detection

A PP2A enzyme activity detection kit (Enzyme-Linked Biotechnology, JK56324), CK enzyme activity detection kit (Enzyme-Linked Biotechnology, JK68421), and PLK enzyme activity detection kit (Enzyme-Linked Biotechnology, JK89575) were used according to the instructions to detect enzyme activities. Briefly, a standard hole, blank hole, and sample hole were set. Then, 50 μL standard was added to the standard well, 50 μL sample dilution buffer to the blank well, and 10 μL sample with 40 μL dilution buffer to the sample well. Then, 100 μL horseradish peroxidase (HRP)-labeled antibody was added to each well. The wells were sealed and incubated at 37 °C for 60 min. The plate was washed five times before 50 μL substrates A and B were added. The plate was incubated at 37 °C for 15 min away from light. Stop solution (50 μL) was added, and the OD value of each well was measured at 450 nm within 15 min. A standard curve with R2 > 0.99 was drawn to prove the existence of a linear relationship. Finally, the concentrations and activities of PP2A, CK, and PLK were calculated.

### Targeted metabolomics

The mouse hippocampus homogenate was melted at 4 °C for 30–60 min. Then, 100 μL homogenate was transferred to a labeled 1.5 mL centrifuge tube, 300 µL methanol added, and the contents mixed on a vortex mixer for 1 h. The precipitate was collected by centrifugation at 12,000 rpm for 10 min. Then, 95 µL supernatant was transferred to a labeled 1.5-mL microcentrifuge tube, and 5 µL of an internal standard of Sph/Cer was added. After mixing well and centrifugation at 12,000 rpm for 10 min, the supernatant was transferred to a 250 µL vial insert for analysis. The ACQUITY UPLC HSS T3 2.1 × 100 mm, 1.8 μm column (Waters, Milford, MA, USA) was used as the stationary phase. It was suited for lipid retention. The mobile phase consisted of solvent A (0.1% formic acid-acetonitrile/water (3:2 v/v)) and solvent B (0.1% formic acid-isopropanol/acetonitrile (9:1 v/v)) with a gradient elution (0–1.5 min, 68–68% A; 1.5–15.5 min, 68–15% A; 15.5–15.6 min, 15–3% A; 15.6–18.0 min, 3–3% A; 18.0–18.1 min, 3–68% A; 18.1–20 min, 68–68% A). The flow rate of the mobile phase was 0.26 mL/min. The column temperature was maintained at 55 °C. The injection volume was 4 μL. A Waters TQ-S mass spectrometer equipped with an ESI probe was employed. The positive ESI source temperature was 150 °C, the capillary voltage was 3.0 kV, the cone voltage was 20 V, the desolvation temperature was 500 °C, and the desolvation gas flow was 1000 L/h. The LC-MS system was controlled by Masslynx software. Data were collected and processed with the same software.

### RT-PCR

An RNeasy kit (Qiagen, 74104) was used to extract total RNA from mice hippocampal tissue, and then the Transcriptor High Fidelity cDNA synthesis kit (Roche, 5081963001) was used to reverse transcribe the RNA into cDNA. All operations were according to the instructions. The following primers were used: mouse β-actin, forward: 5′-GGCTGTATTCCCCTCCATCG-3′, reverse: 5′-CCAGTTGGTAACAATGCCATGT-3′; mouse Acer2, forward: 5′-TGGTGCGAGGACAACTACACTATC-3′, reverse 5′-AAGCATCTGACCCAGGAAACTC-3′.

### Primary neuron culture and processing

A newborn mouse was disinfected and its head was cut off immediately. The cerebral cortex was removed and the hippocampus was separated under a stereomicroscope. The tissue was cut to <1 mm^3^ in DMEM medium (Gibco, C11995500B7). A 1:40 volume ratio of papain solution was added with subsequent digestion at 37 °C for 30 min. The papain was removed, DMEM culture medium was added, and the tissue was blown to make a cell suspension. The cell suspension was passed through a 40 μm filter (Falcon, 352340). The cells were diluted to a certain density and then moved to polylysine (Sigma, P1399)-coated plates for culturing at 37 °C in a 5% CO_2_ incubator. After 4–8 h, DMEM was replaced with Neurobasal medium (Gibco, 21103049) containing 50 × B27 (Gibco, 17504044), 100× glutamine (Invitrogen, 25030081), and 100× penicillin-streptomycin (Thermo, 15140122). Half of the medium was changed every 3 days after cell adhesion.

### Cell viability detection

Cell viability was detected by the MTT assay kit (Sigma, M2128). MTT (5 mg) was placed in a 5 mL EP tube, and PBS buffer (1 mL) added to dissolve the MTT. After blending, the stock solution was kept at −20 °C and protected from light. After the experimental treatments, the cell supernatant in the 96-well plate was discarded, 10 μL MTT solution and 90 μl culture solution were added to the pre-designed cell wells. The cells were incubated at 37 °C with 5% CO_2_ for 4 h. The supernatant was discarded and 100 μl DMSO was added and mixed gently for 10 min while avoiding light. The OD value at 490 nm was detected by an enzyme marker.

### Cytotoxicity detection

Cytotoxicity was detected by the LDH assay (Roche, 4744926001). The powder was dissolved in ddH_2_O and mixed thoroughly to make the catalytic solution. Then, 250 μL of the catalytic solution was added to the staining solution (11.25 mL) and mixed thoroughly. Then, 100 μL cell supernatant of each group was added to the new 96-well plate. The LDH reaction solution (100 μL) was added with subsequent incubation at room temperature away from light for 30 min. After the incubation, 50 μL stop solution was added to each well and gently mixed for 10 min. The OD value of each well was measured at 490 nm by a microplate reader.

### Cell death detection

PI/Hoechst detection was used to detect the cell death rate. Hoechst labels all cells as blue fluorescence, and PI labels only dead cells as red fluorescence. Therefore, the ratio of red to blue can be used to calculate the cell death rate. After the cells were treated, the original medium was discarded, and the cells were rinsed three times with PBS. The PI (Sigma, P4170) and Hoechst (Sigma, B2261) mixture was added into the cell culture well and incubated at 37 °C for 10 min under dark conditions. The cells were removed from the incubator, the mixture of PI and Hoechst was discarded, and the cells were rinsed three times with PBS. Confocal microscopy was used for observation and imaging.

### Statistical analysis

Excel and GraphPad Prism 9.0 software were used for data preservation, recording, statistics, and analyses. Image data were analyzed by ImageJ and other software. All results were analyzed using the *t*-test, one-way ANOVA, and two-way ANOVA as appropriate. Data are expressed as the mean ± standard error (mean ± SEM), with *P* ≤ 0.05 as a significant difference. In animal behavioral tests, *n* ≥ 10; in protein detection experiments, such as western blot and immunofluorescence, *n* ≥ 3.

## Supplementary information

Supplemental material

Original Data File

## Data Availability

All data generated or analyzed during this study are included in this published article and its supplementary information files.
