# Supplementary material for: Chronic hypoxia leads to cognitive impairment by promoting HIF-2α-mediated ceramide catabolism and alpha-synuclein hyperphosphorylation
Source: Cell Death Discov. 2022 Nov 30;8:473. doi: 10.1038/s41420-022-01260-6 (PMC9712431; doi:10.1038/s41420-022-01260-6)
Supplement: Supplementary file 1 — Supplemental material [file 41420_2022_1260_MOESM1_ESM.docx]

**
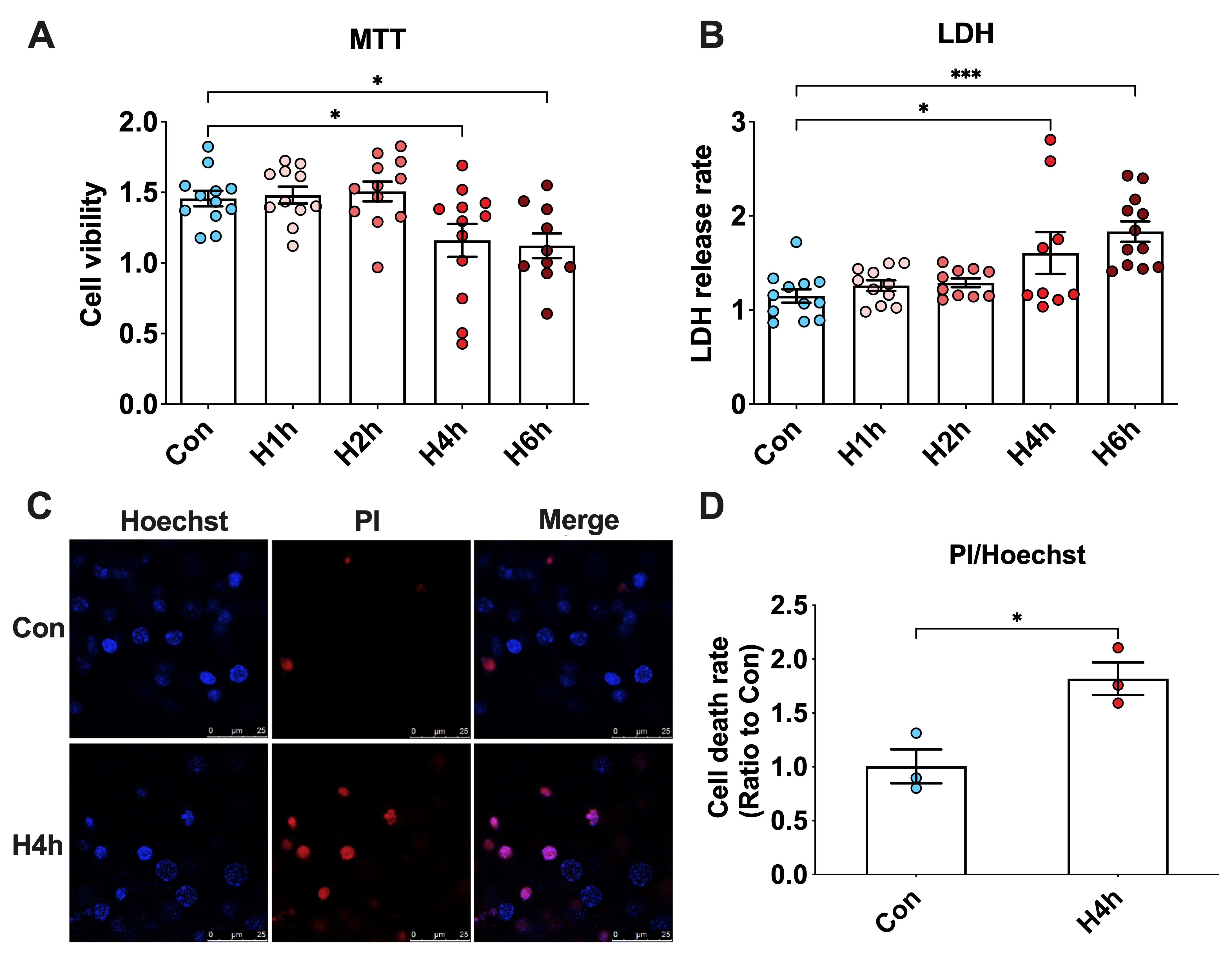
**

**Supplemental Figure 1. Persistent hypoxia induces neuronal cytotoxicity.** Mouse hippocampal neurons were cultured and treated with continuous hypoxic conditions for different periods (0, 1, 2, 4, 6 h). A. The cell viability of neurons was evaluated by the MTT cell viability assay. B. The cytotoxicity of neurons was evaluated by the LDH assay. C. The cell mortality of neurons was detected by PI/Hoechst co-staining; Statistical analysis was performed. In A–B, data are expressed as the mean ± SEM (one-way ANOVA), ^*^P<0.05, ^***^P<0.001, n = 4. In D, data are expressed as the mean ± SEM (unpaired *t*-test), ^*^P<0.05, n = 3.

**
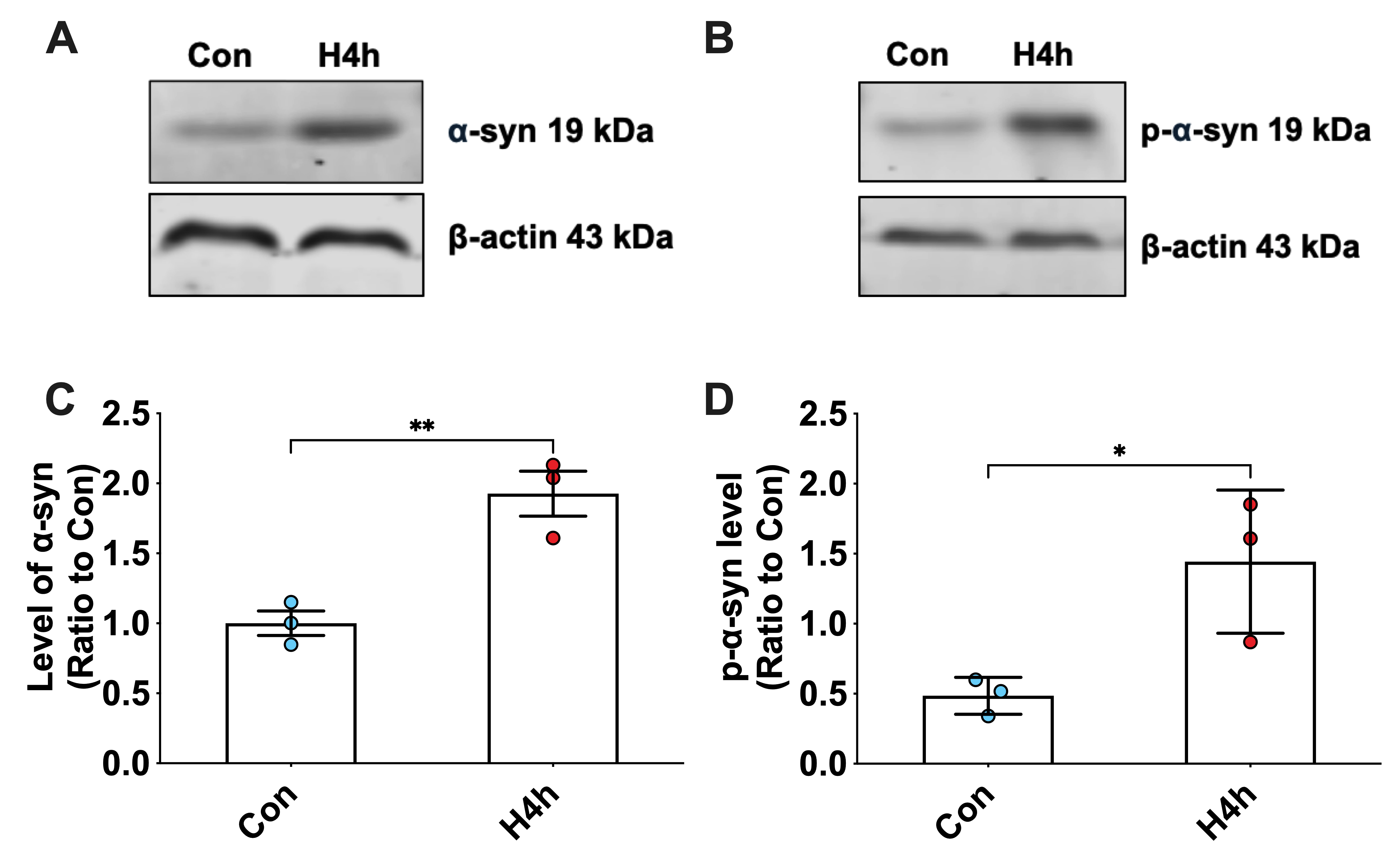
**

**Supplemental Figure 2. Persistent hypoxia increases the levels of α-syn and its phosphorylation in primary neurons.** A–D. Mouse hippocampal neurons were cultured and treated under continuous hypoxic conditions for 4 h. The levels of p-α-syn and α-syn were detected by western blots with β-actin as the internal reference; Statistical analysis was performed. Data are expressed as the mean ± SEM (unpaired *t*-test), ^*^P<0.05, ^**^P<0.01, n = 3.

**
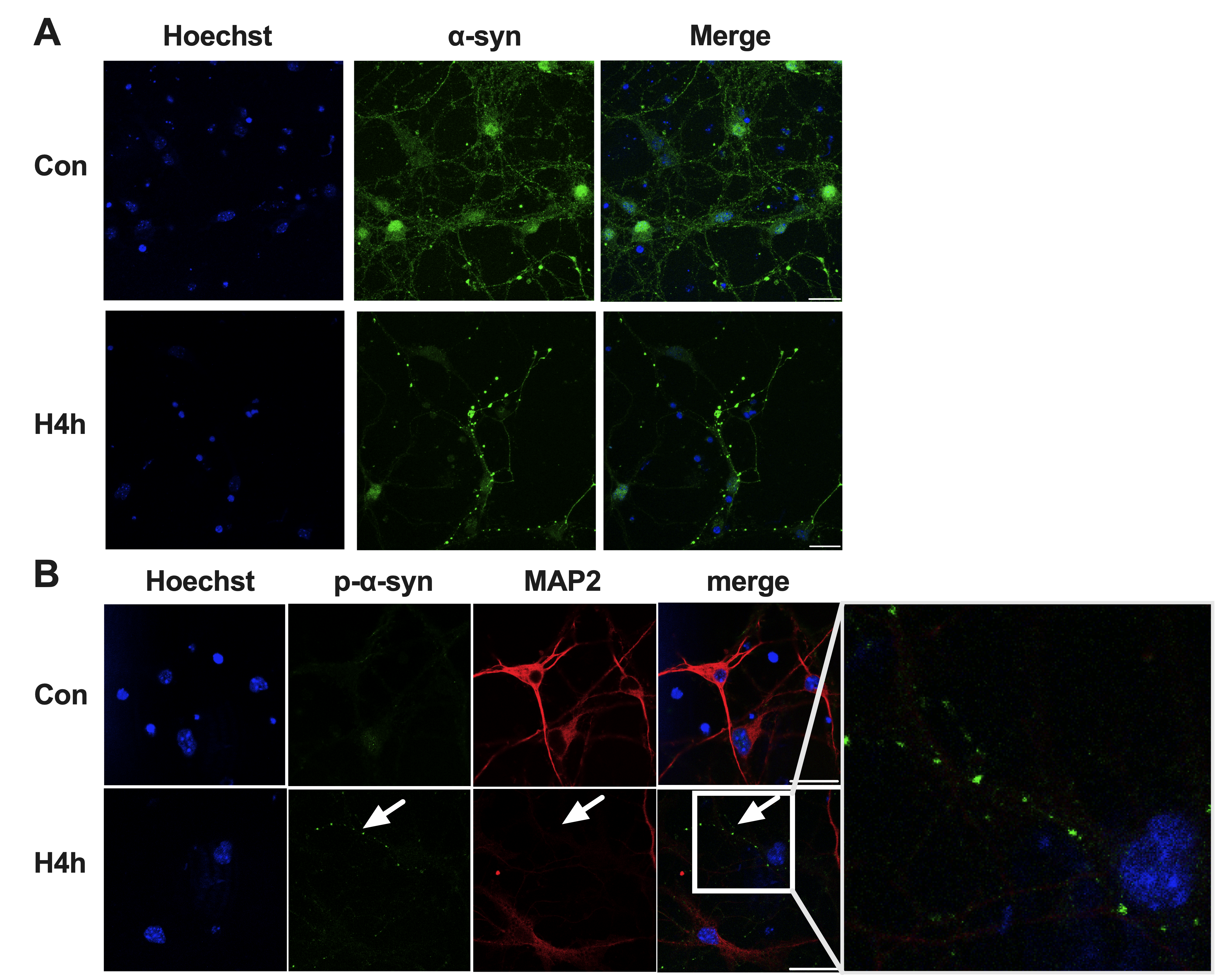
**

**Supplemental Figure 3. Persistent hypoxia induces phosphorylation and aggregation of α-syn.** A–B. Mouse hippocampal neurons were cultured and treated under continuous hypoxic conditions for 4 h. Immunofluorescence staining was performed for α-syn and p-α-syn, Map2-labeled neurons, and Hoechst-labeled nuclei. Bar = 25 μm.


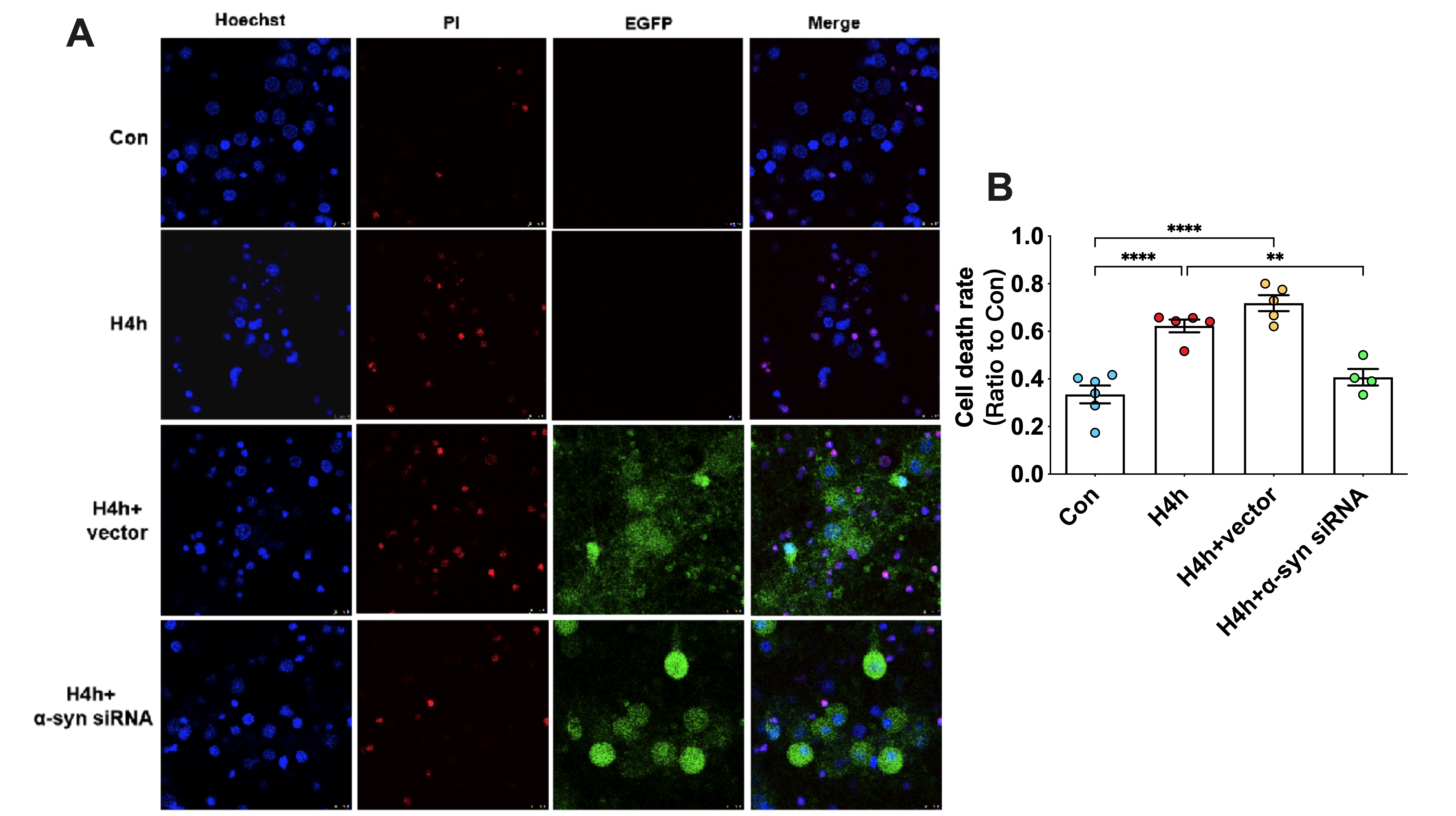


**Supplemental Figure 4. α-Syn knockdown alleviates neuronal injury induced by persistent hypoxia.** A–B. Mouse hippocampal neurons were treated with α-syn siRNA or vector lentivirus (with EGFP), and then kept under continuous hypoxic conditions for 4 h. The cell mortality of neurons was detected by PI/Hoechst co-staining; Statistical analysis was performed. Data are expressed as the mean ± SEM (one-way ANOVA), ^**^P<0.01, ^****^P<0.0001, n = 4–5.


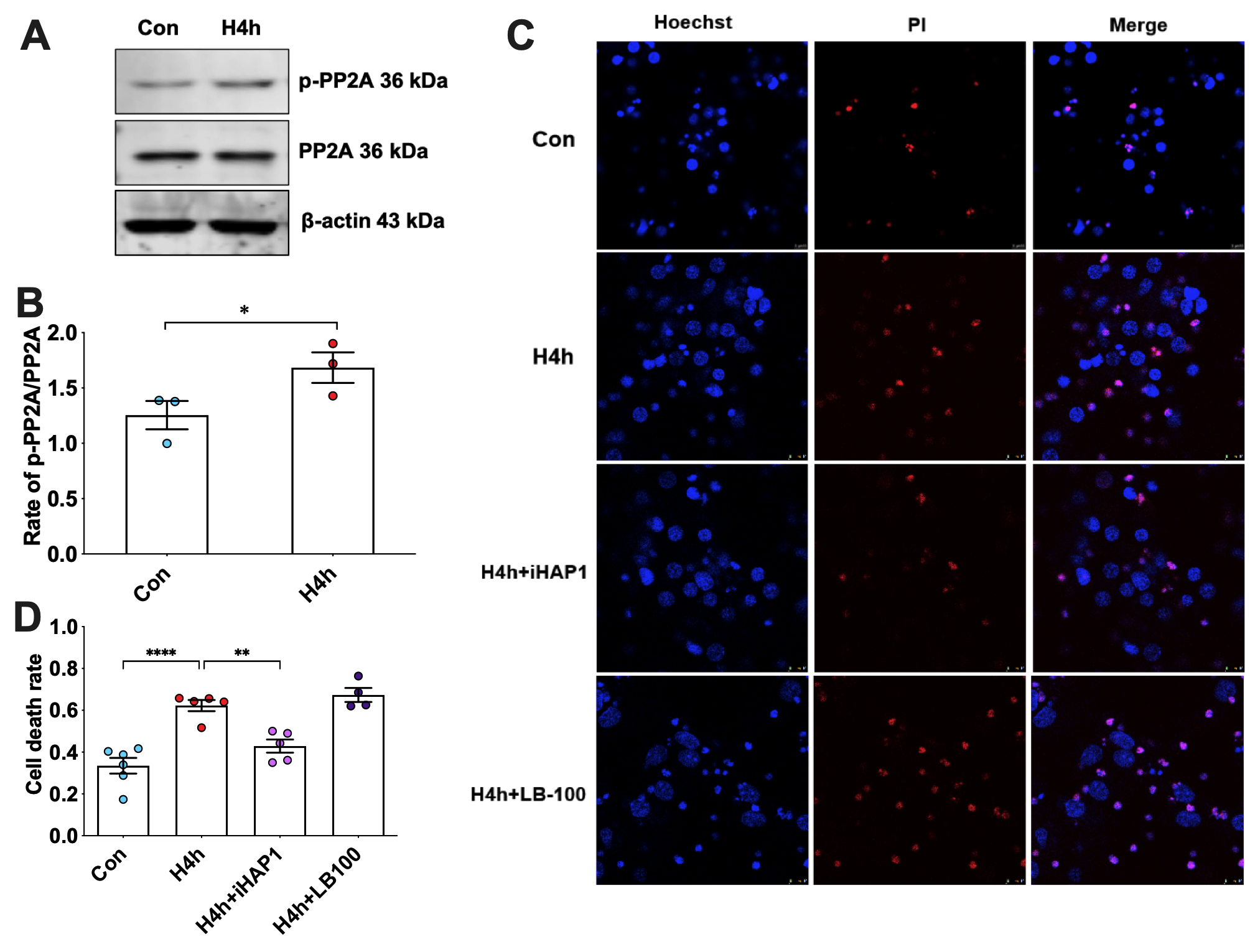


**Supplemental Figure 5. PP2A activation alleviates neuronal injury induced by persistent hypoxia.** A–B. Mouse hippocampal neurons were cultured and treated with continuous hypoxic conditions for 4 h. The levels of p-PP2A and PP2A were detected by western blots with β-actin as the internal reference; statistical analysis was performed. C, D. Primary mouse neurons were treated with iHAP1, a PP2A agonist, and LB-100, a PP2A inhibitor, and then kept under continuous hypoxic conditions for 4 h. The cell mortality of neurons was detected by PI/Hoechst co-staining; statistical analysis was performed. In B, data are expressed as the mean ± SEM (unpaired *t*-test), ^*^P<0.05, n = 3. In D, data are expressed as the mean ± SEM (One-way ANOVA), ^**^P<0.01, ^****^P<0.0001, n = 4–5.
