# Supplementary material for: Chronic hypoxia leads to cognitive impairment by promoting HIF-2α-mediated ceramide catabolism and alpha-synuclein hyperphosphorylation
Source: Cell Death Discov. 2022 Nov 30;8:473. doi: 10.1038/s41420-022-01260-6 (PMC9712431; doi:10.1038/s41420-022-01260-6)
Supplement: Supplementary file 2 — Original Data File [file 41420_2022_1260_MOESM2_ESM.docx]

Original western blots

Figure2A


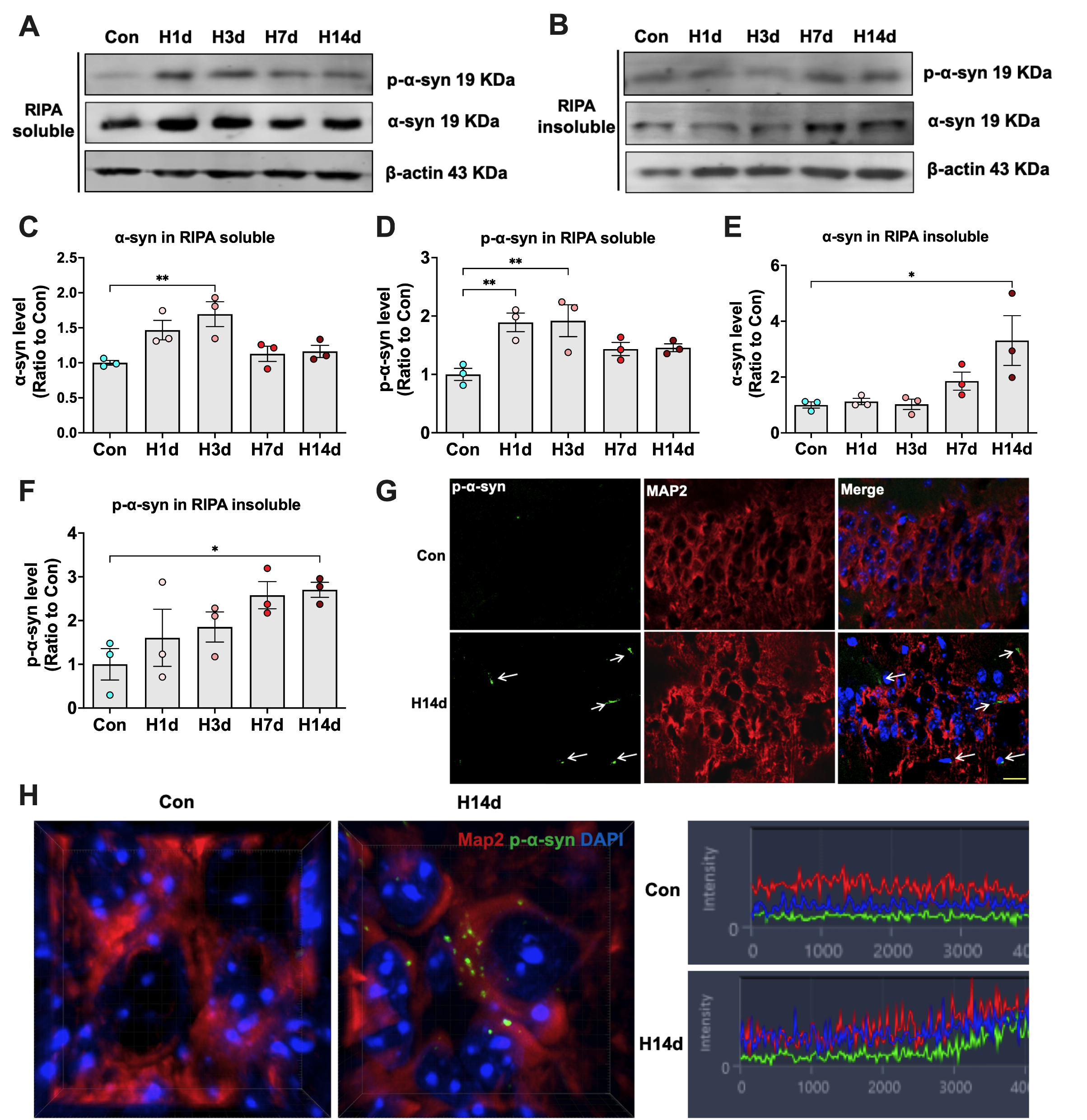


Original western blots


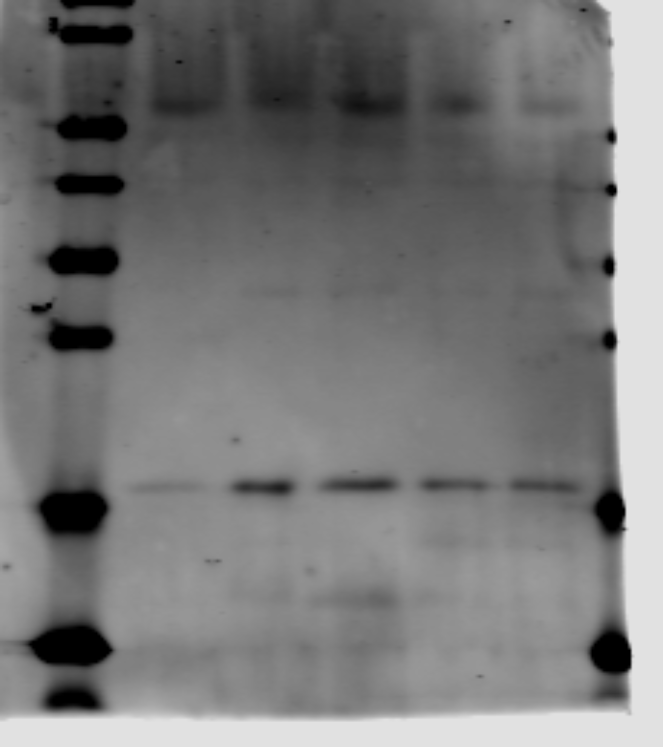


p-α-syn


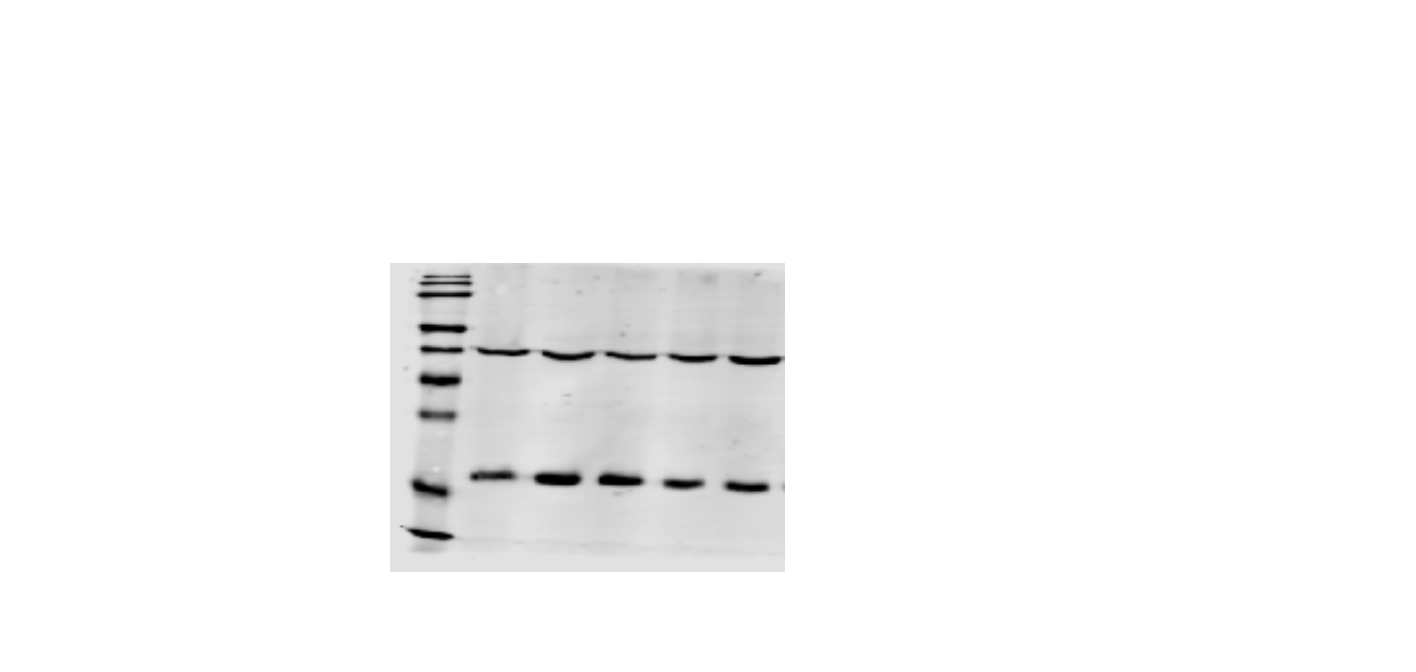


β-actin

α-syn

Figure2B


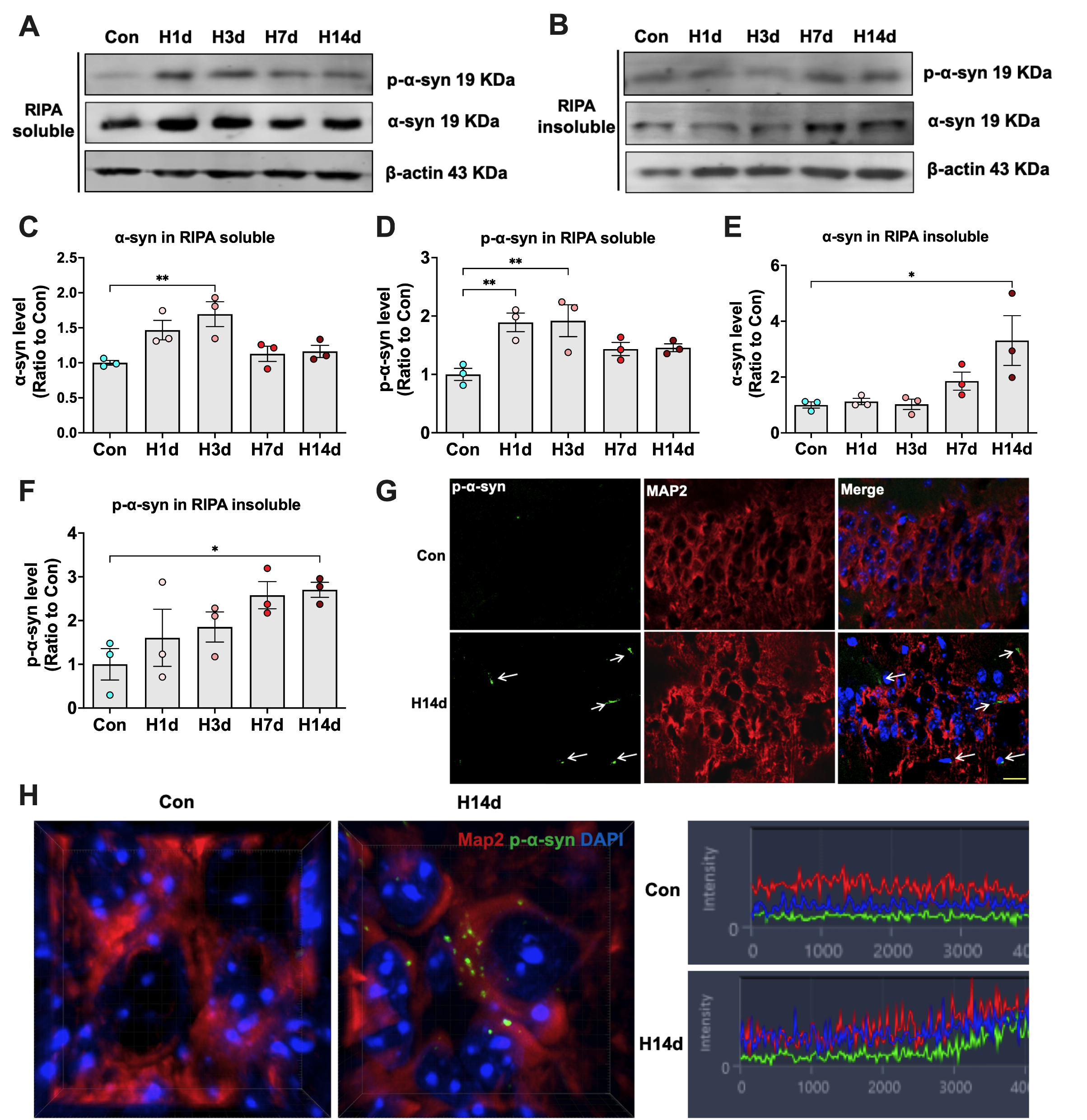


Original western blots


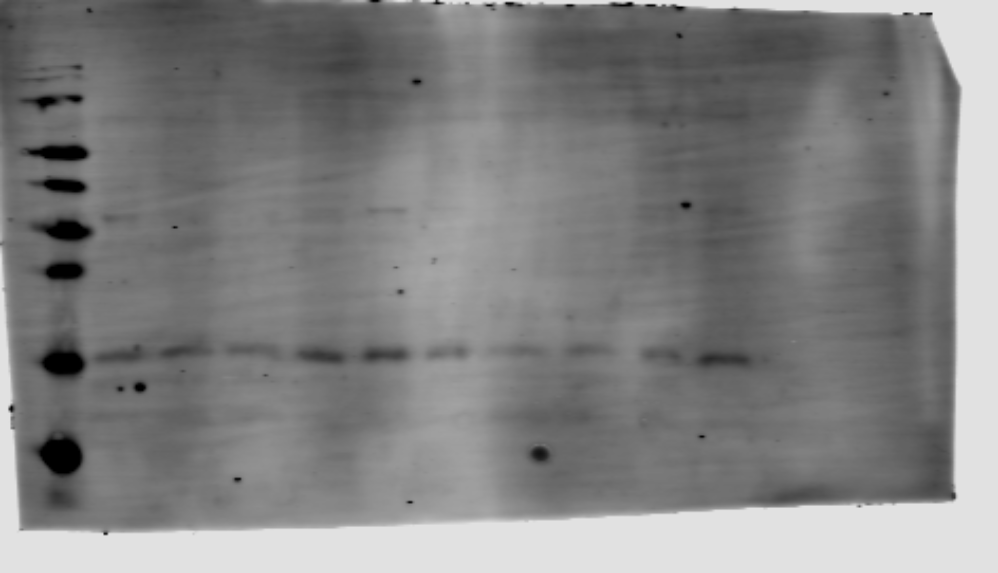


p-α-syn


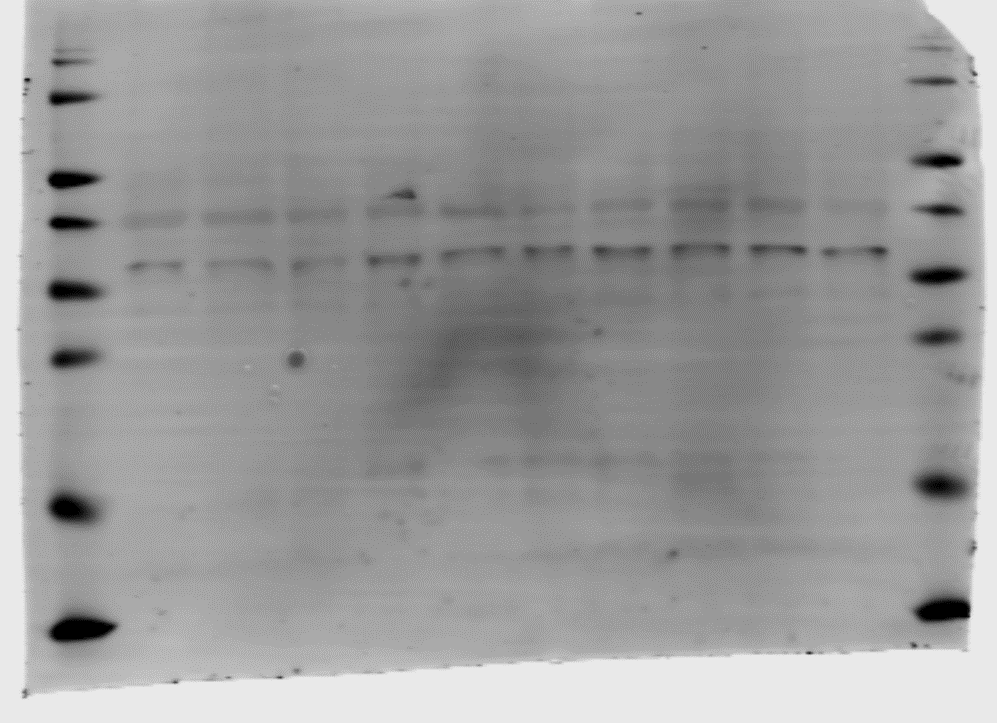


α-syn


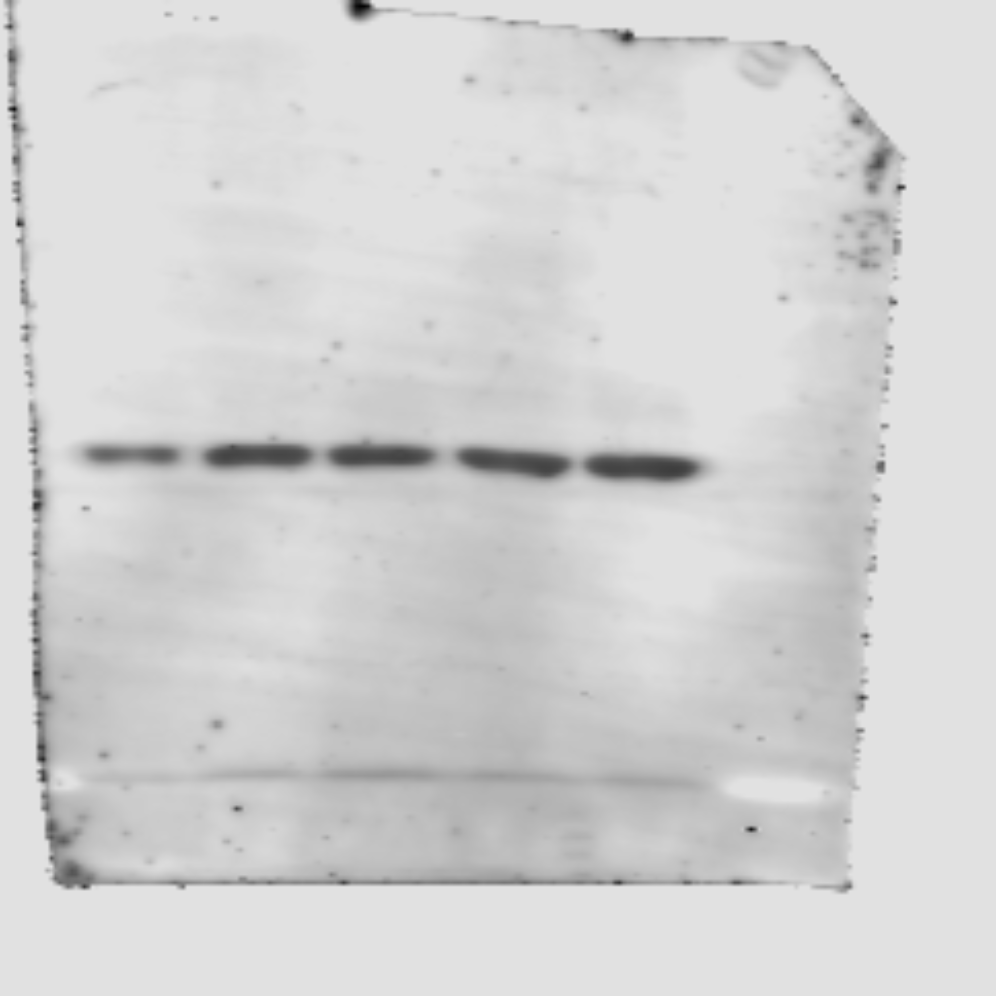


β-actin

Figure3B


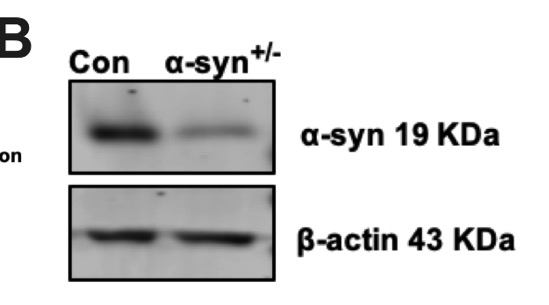


Original western blots


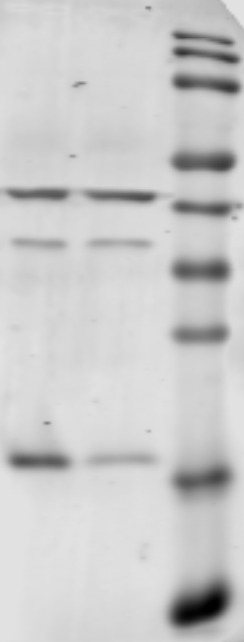


β-actin

α-syn

Figure3F


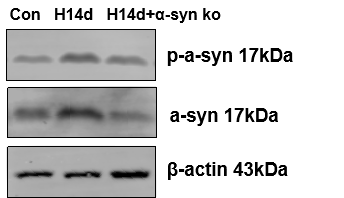


Original western blots


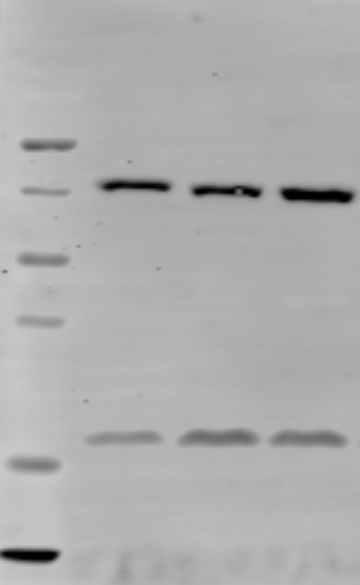


β-actin

p-α-syn


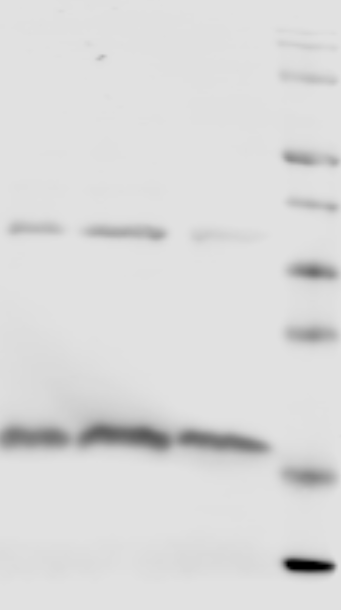


α-syn

Figure4B


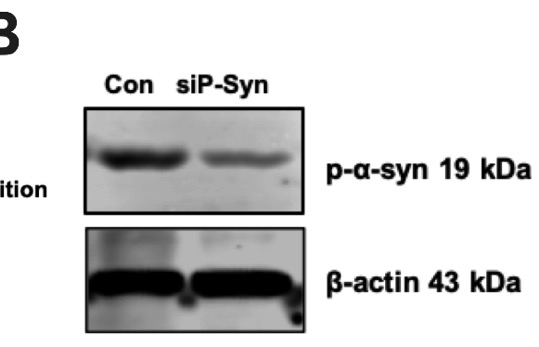


Original western blots


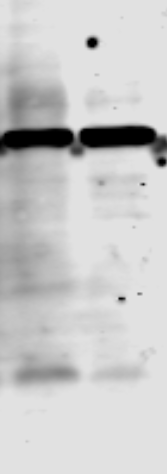


p-α-syn

β-actin

Figure4F


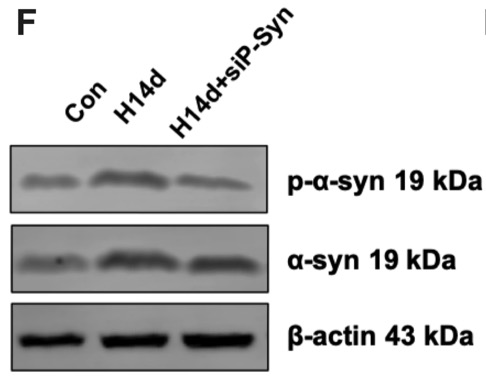


Original western blots


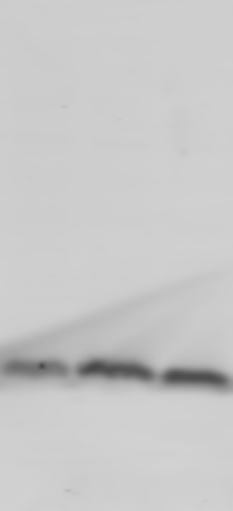


p-α-syn


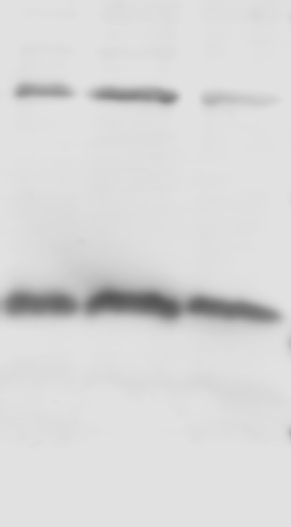


α-syn


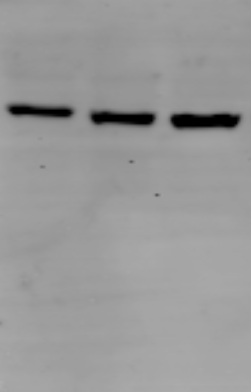


β-actin

Figure5D


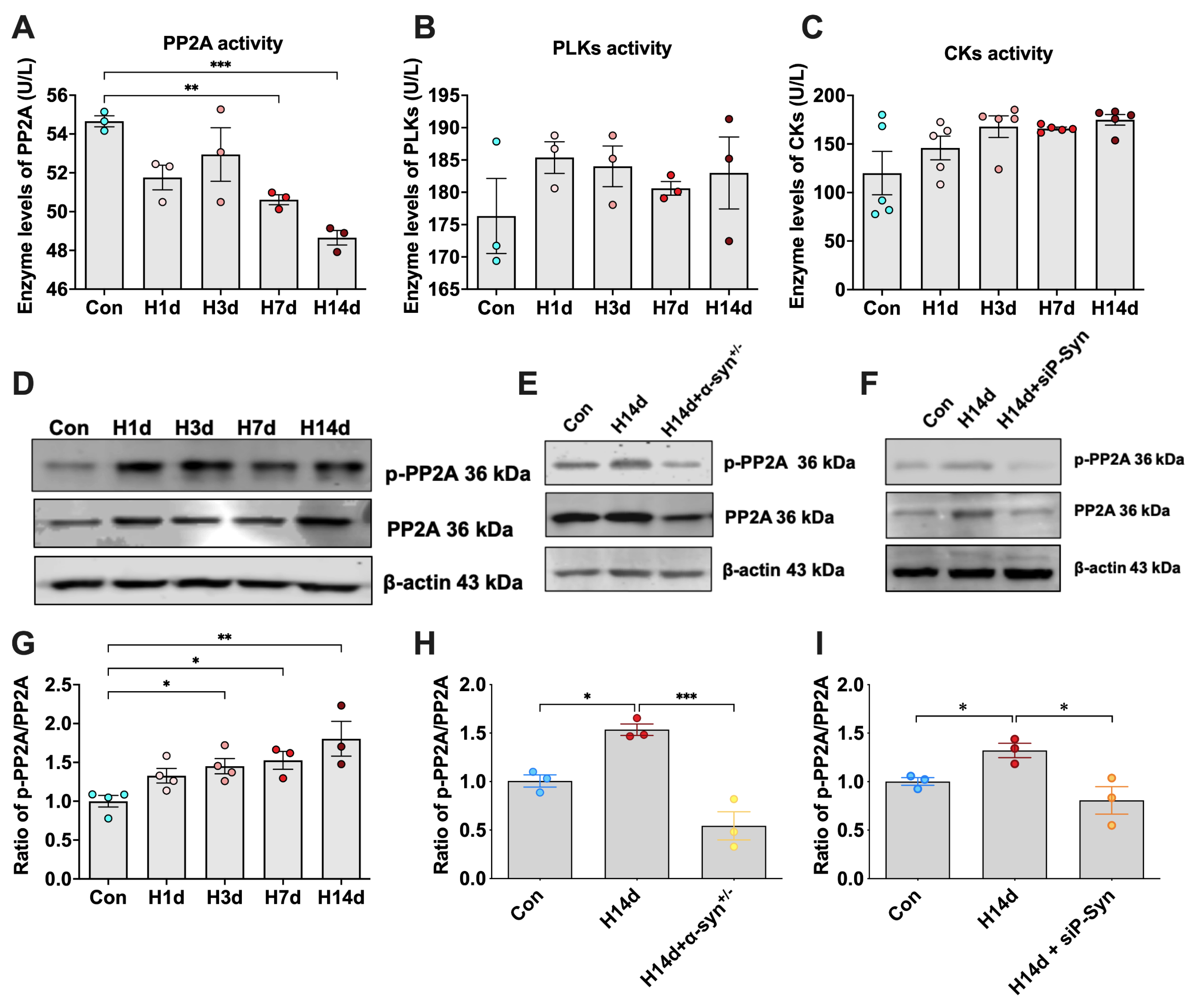


Original western blots


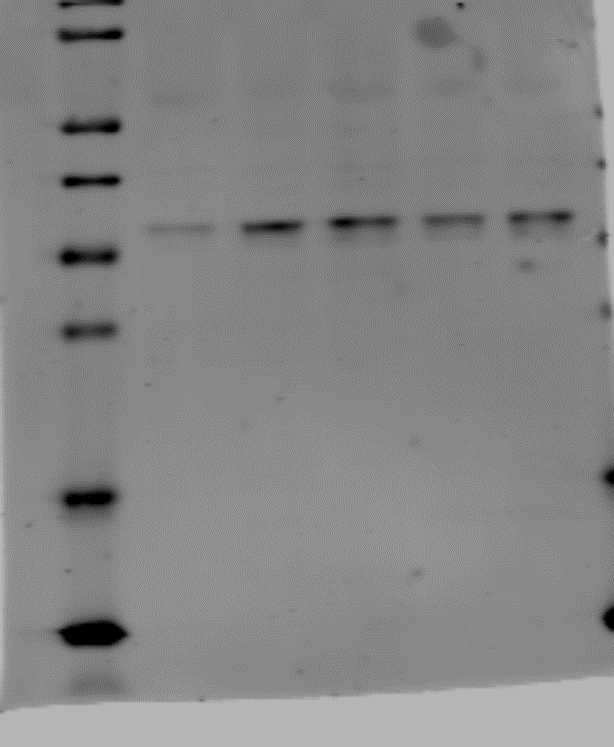


p-PP2A


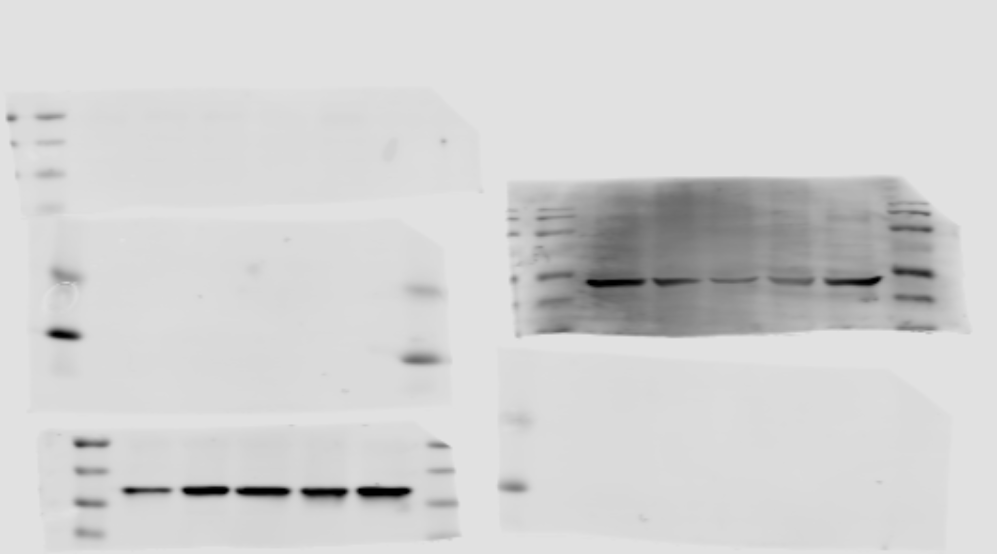


PP2A


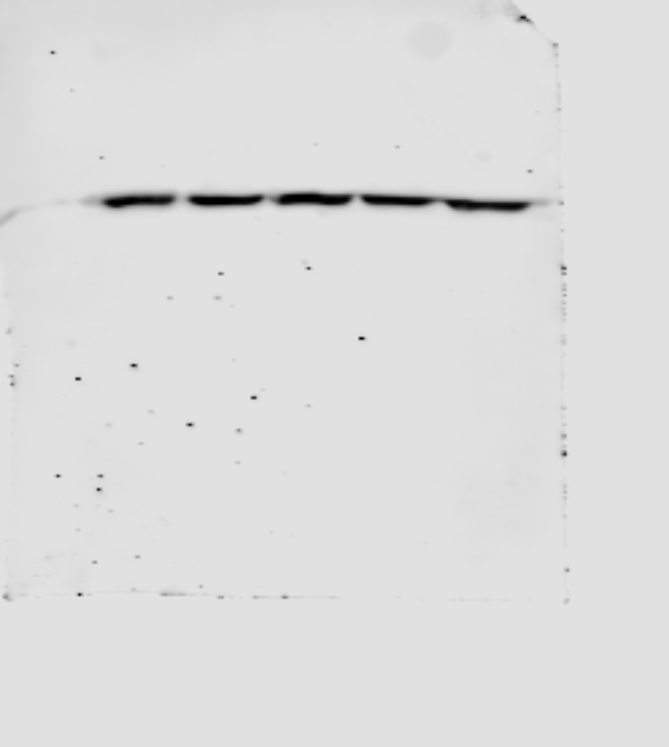


β-actin

Figure5E


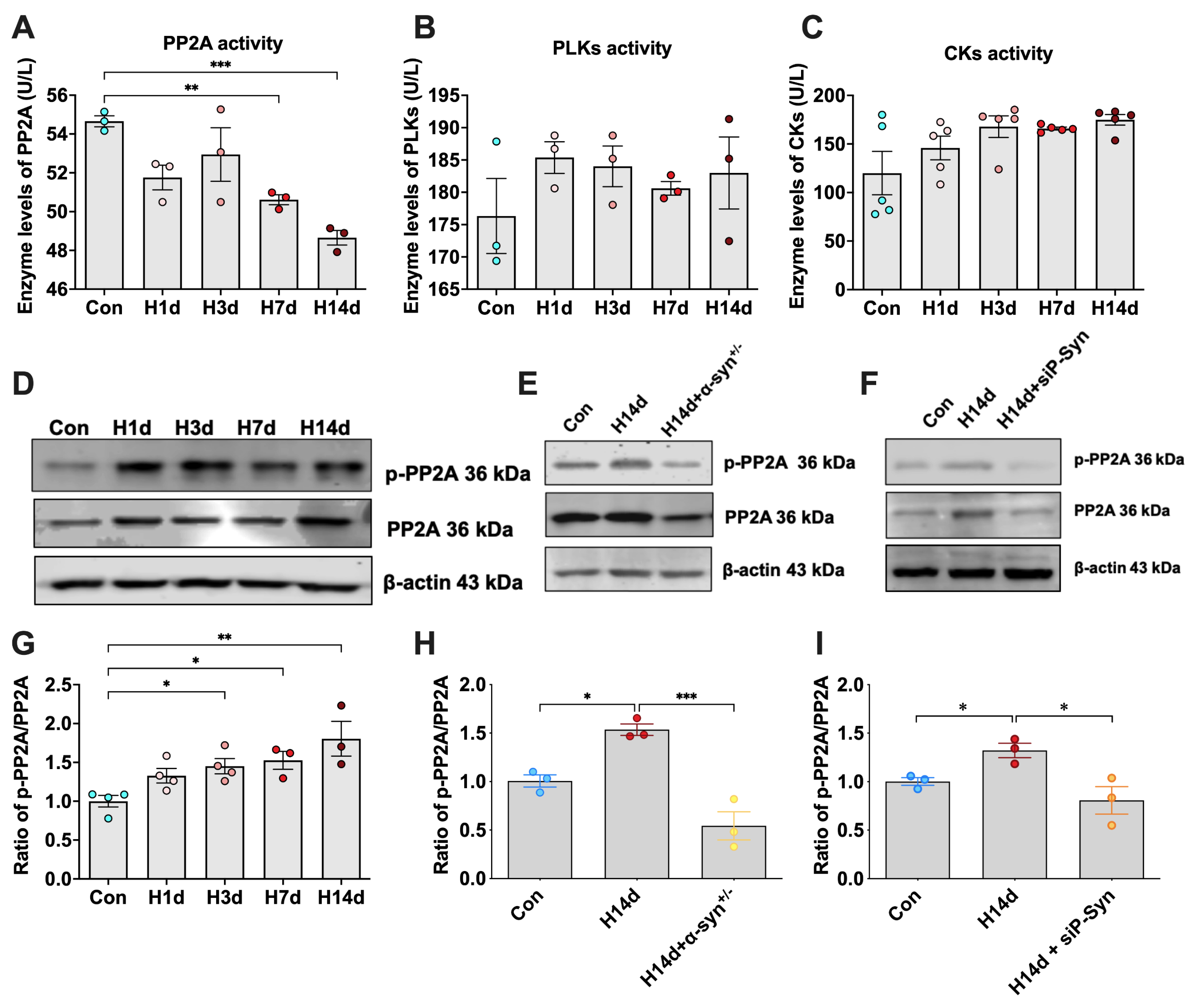


Original western blots


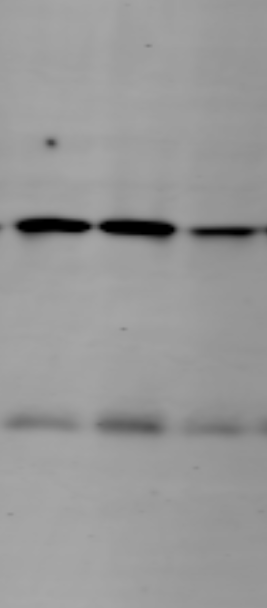


PP2A


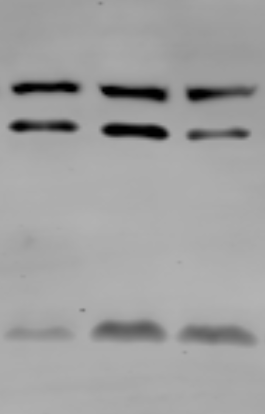


β-actin

p-PP2A

Figure5F


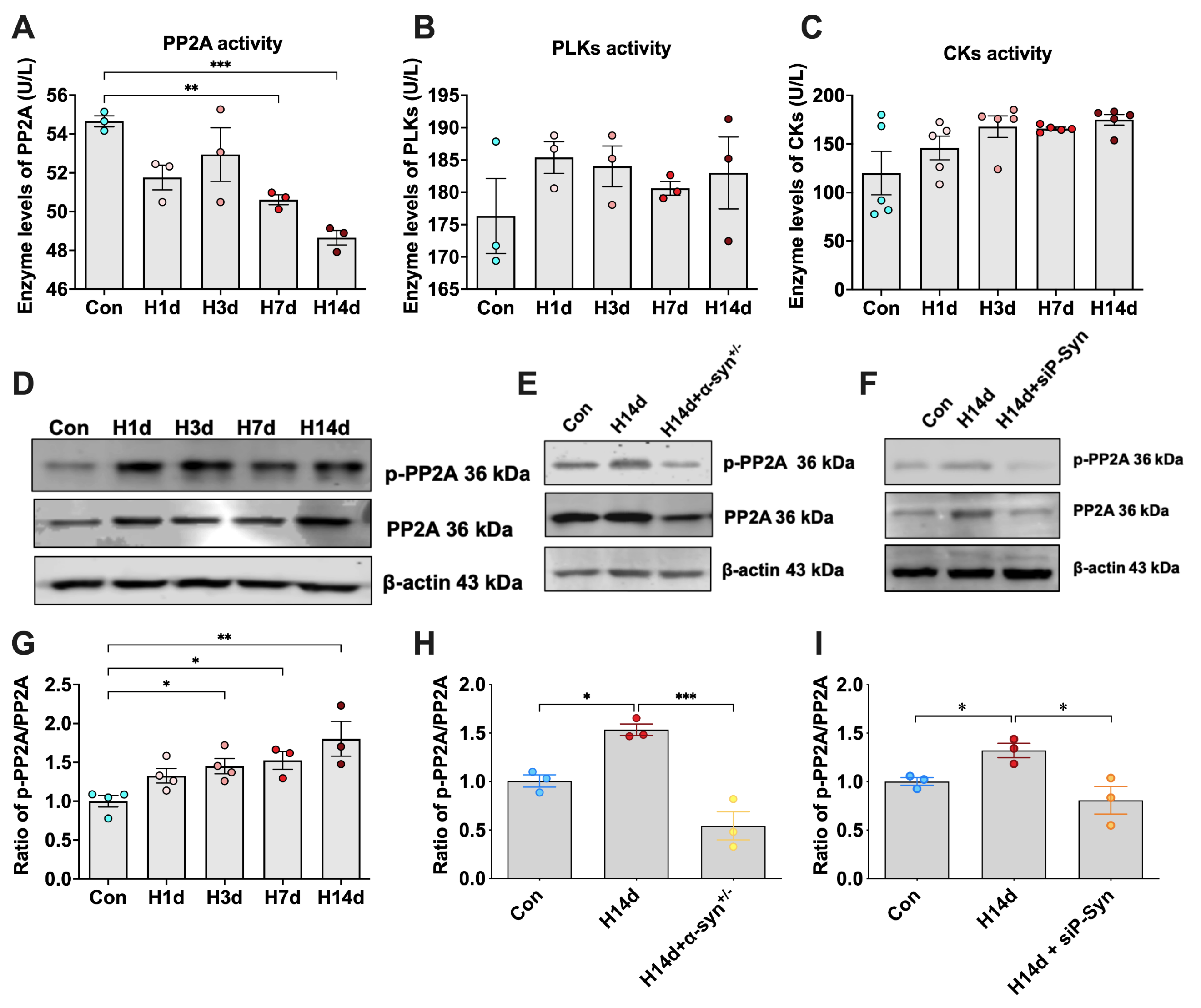


Original western blots


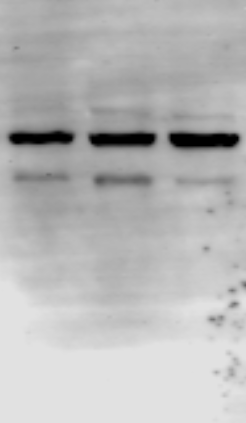


PP2A

β-actin


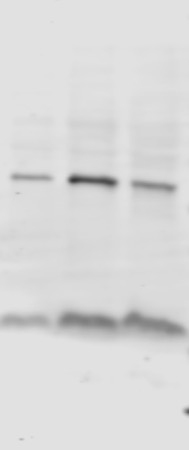


p-PP2A

Figure6F


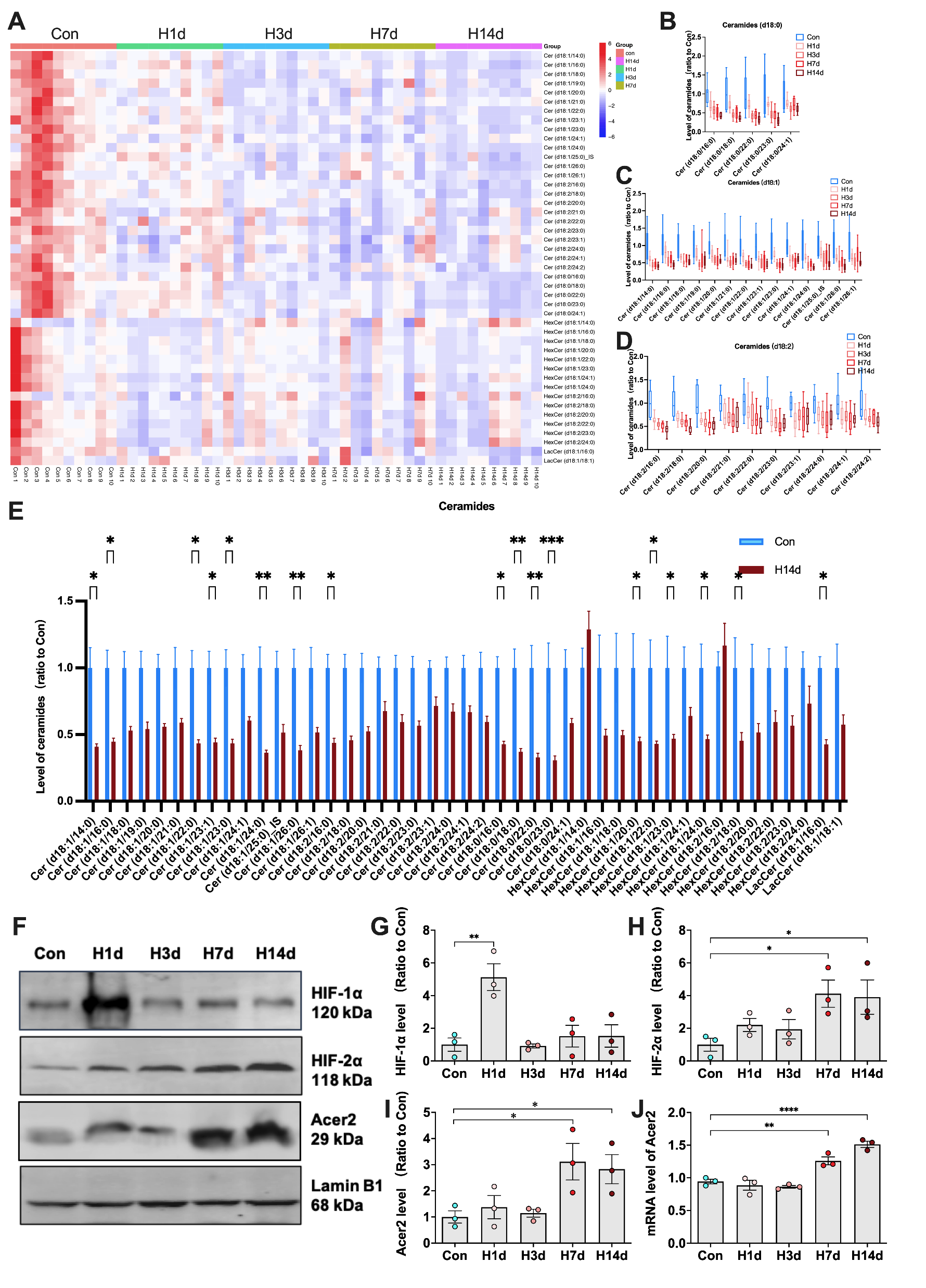


Original western blots


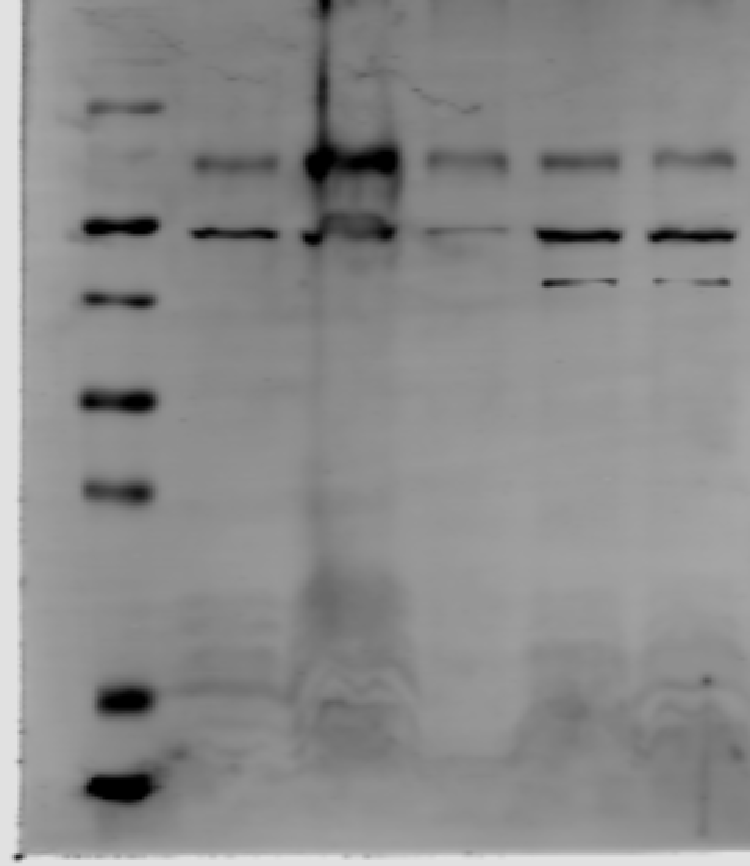


HIF-1α


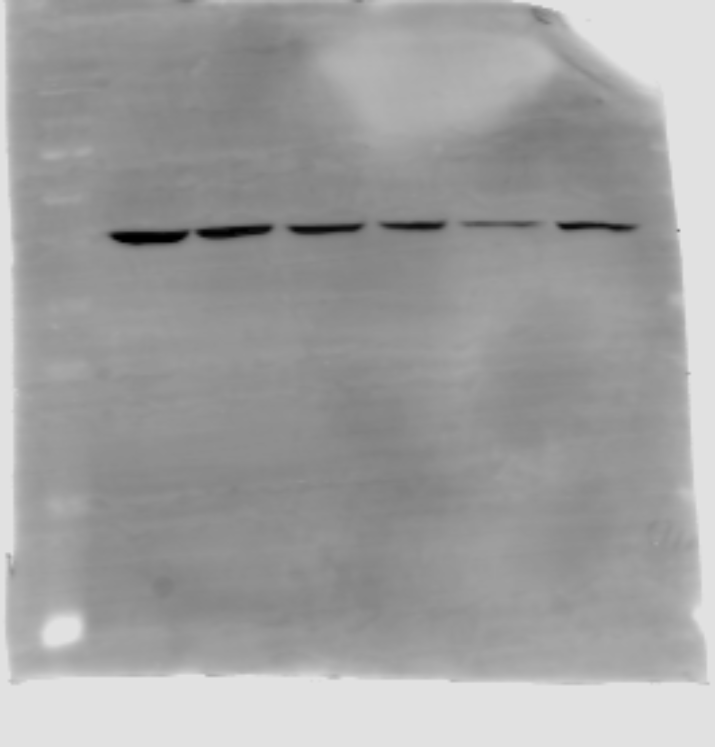


HIF-2α


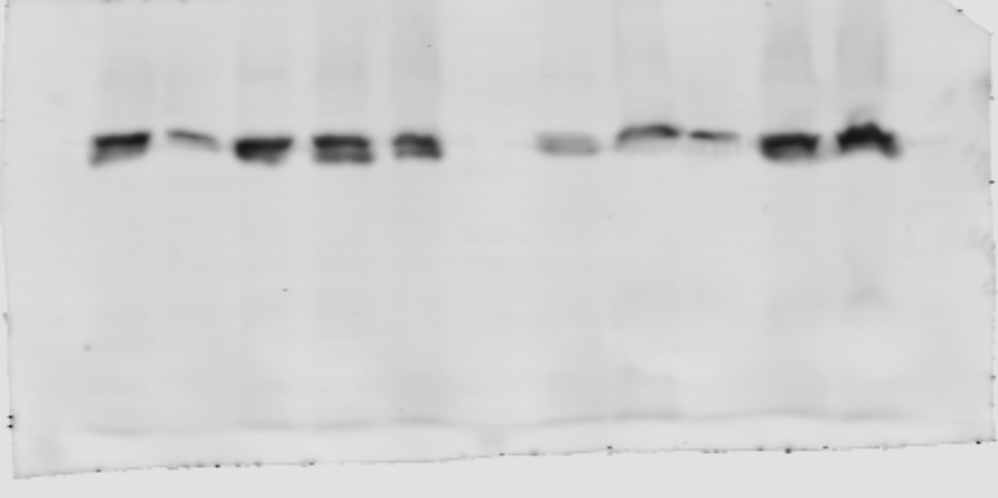


Acer2


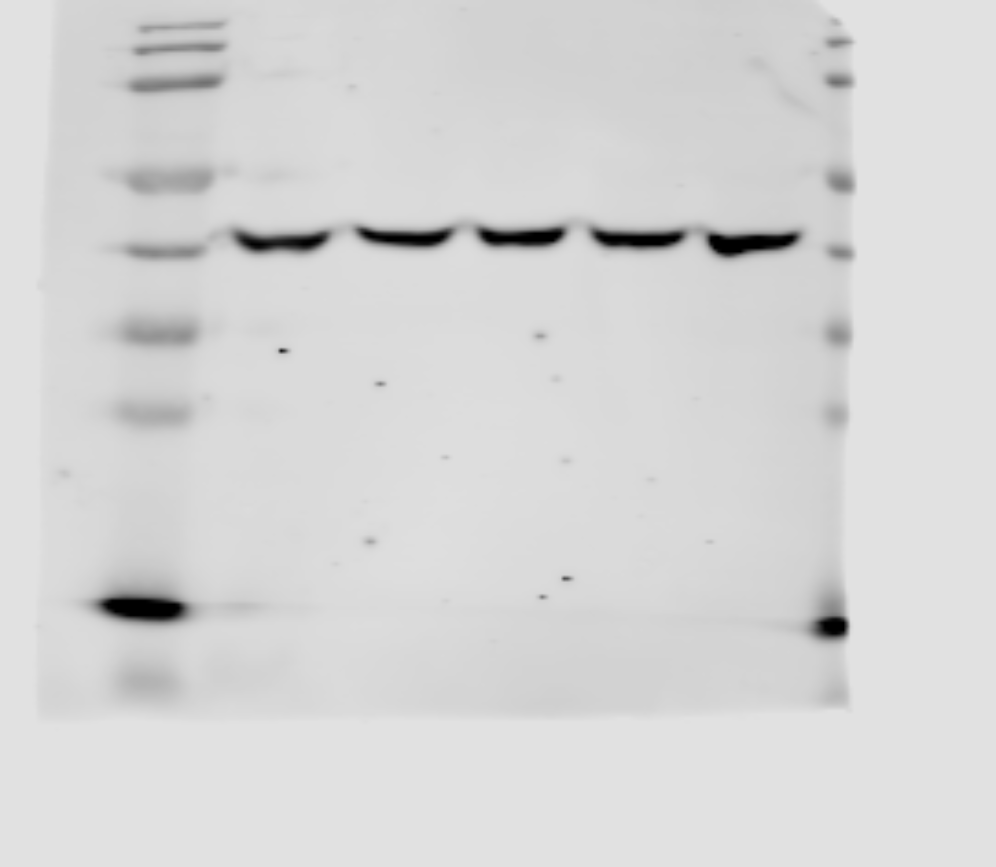


LaminB1

Figure7G


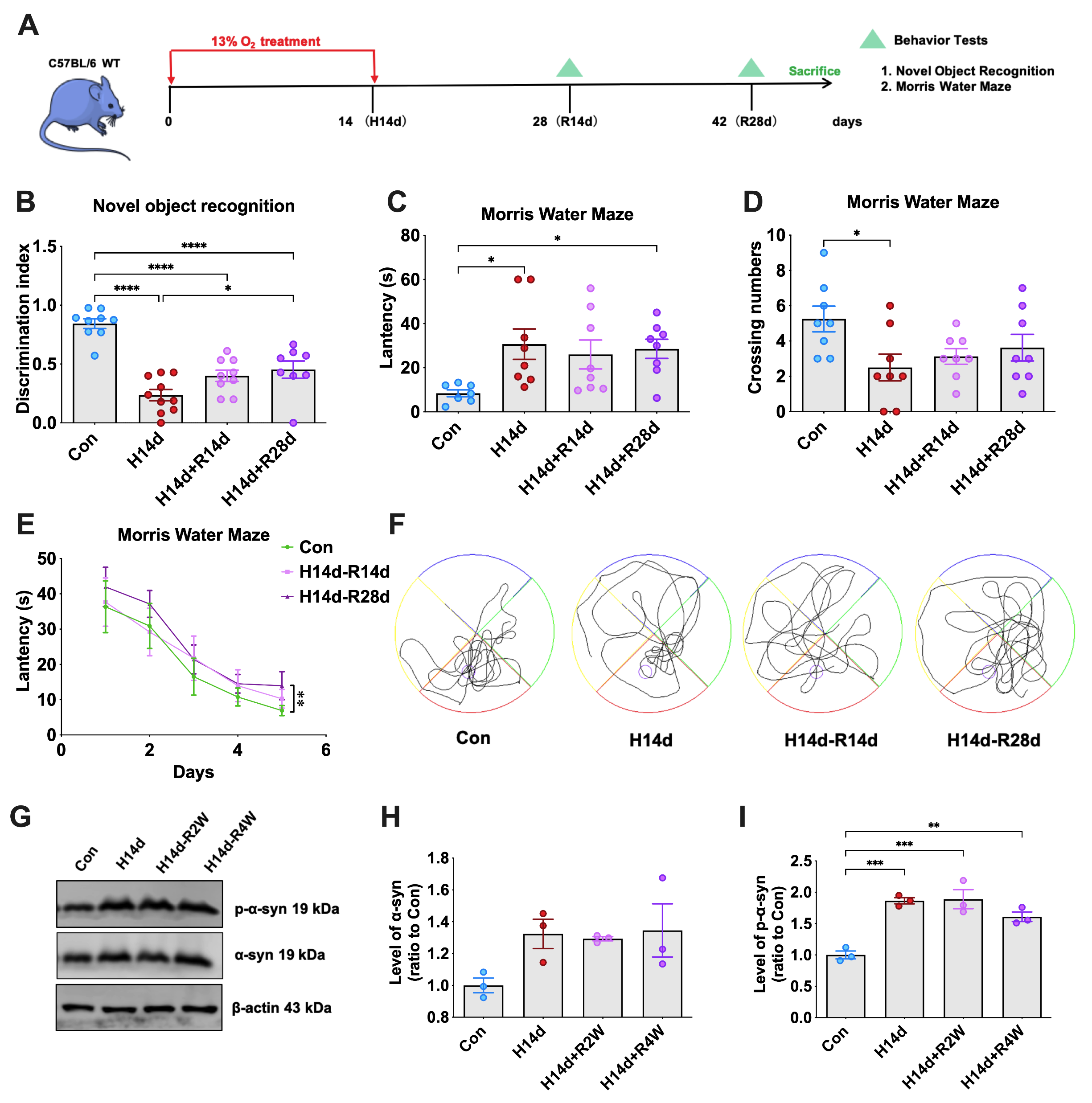


Original western blots


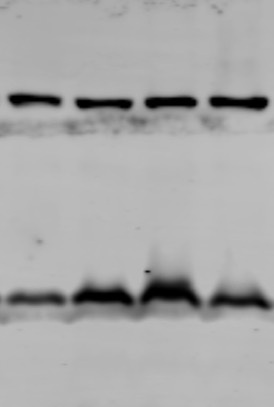


p-α-syn

β-actin


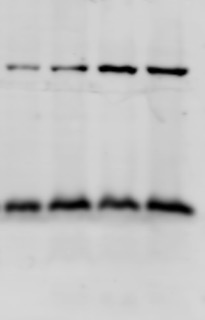


α-syn

Figure8H


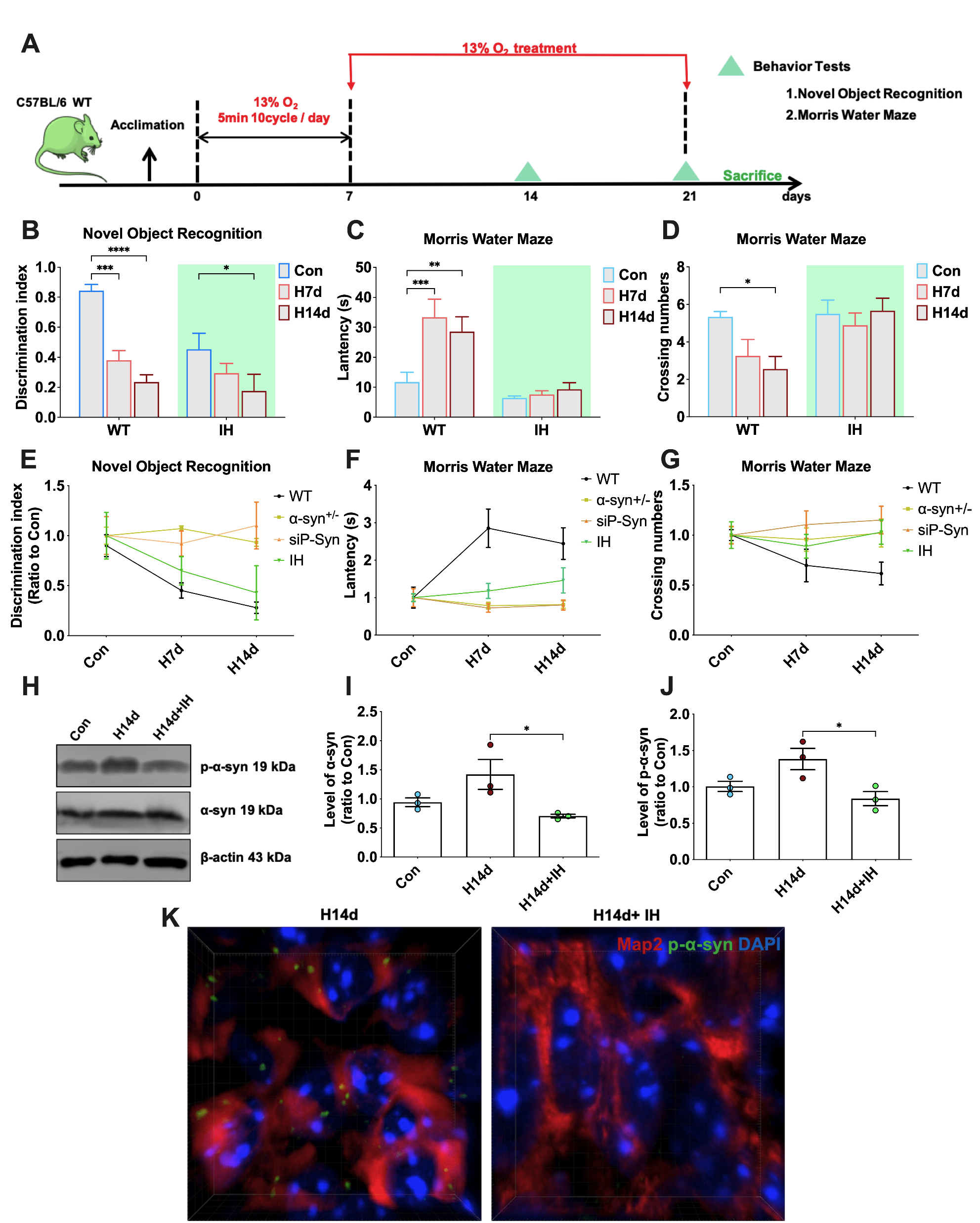


Original western blots


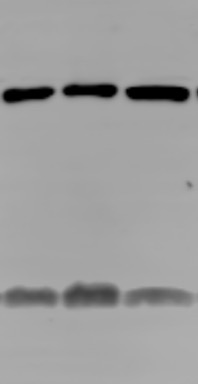


p-α-syn

β-actin


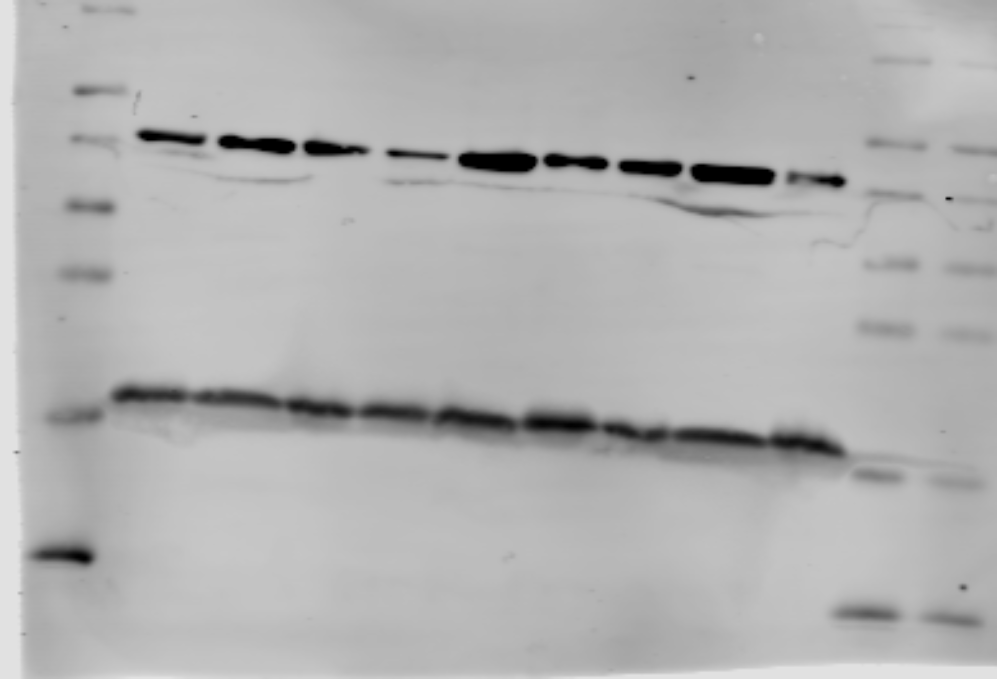


α-syn

Figure S2A


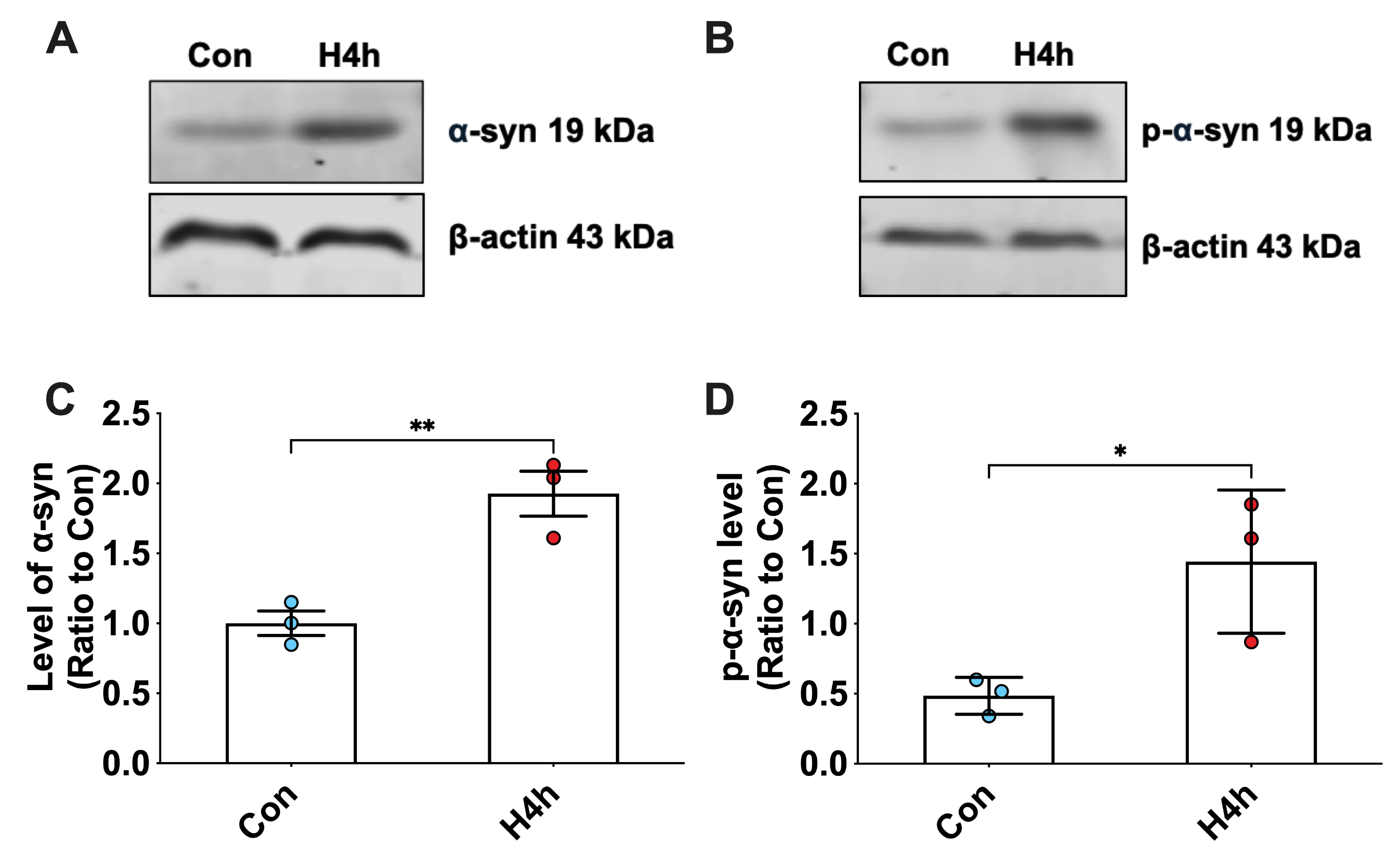


Original western blots


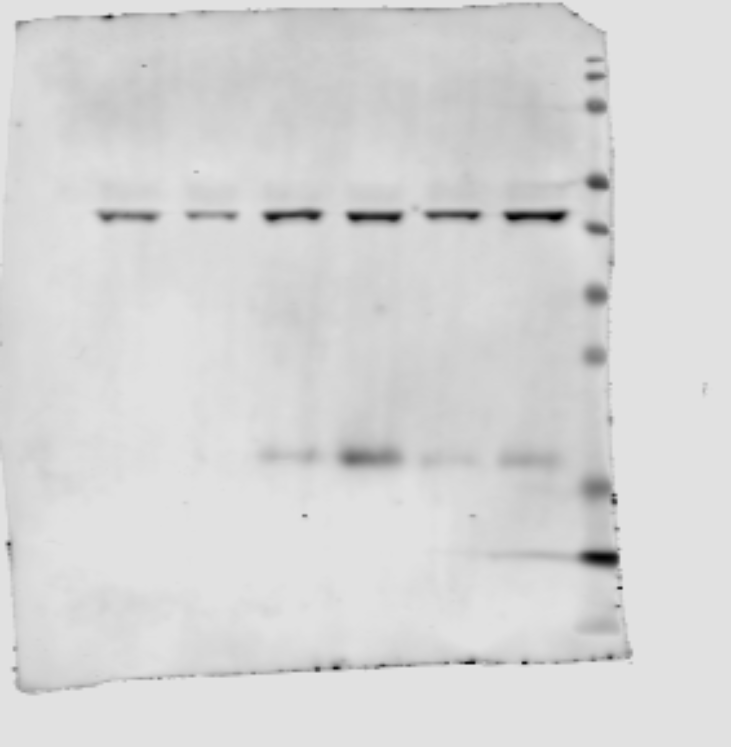


α-syn

β-actin

Figure S2B


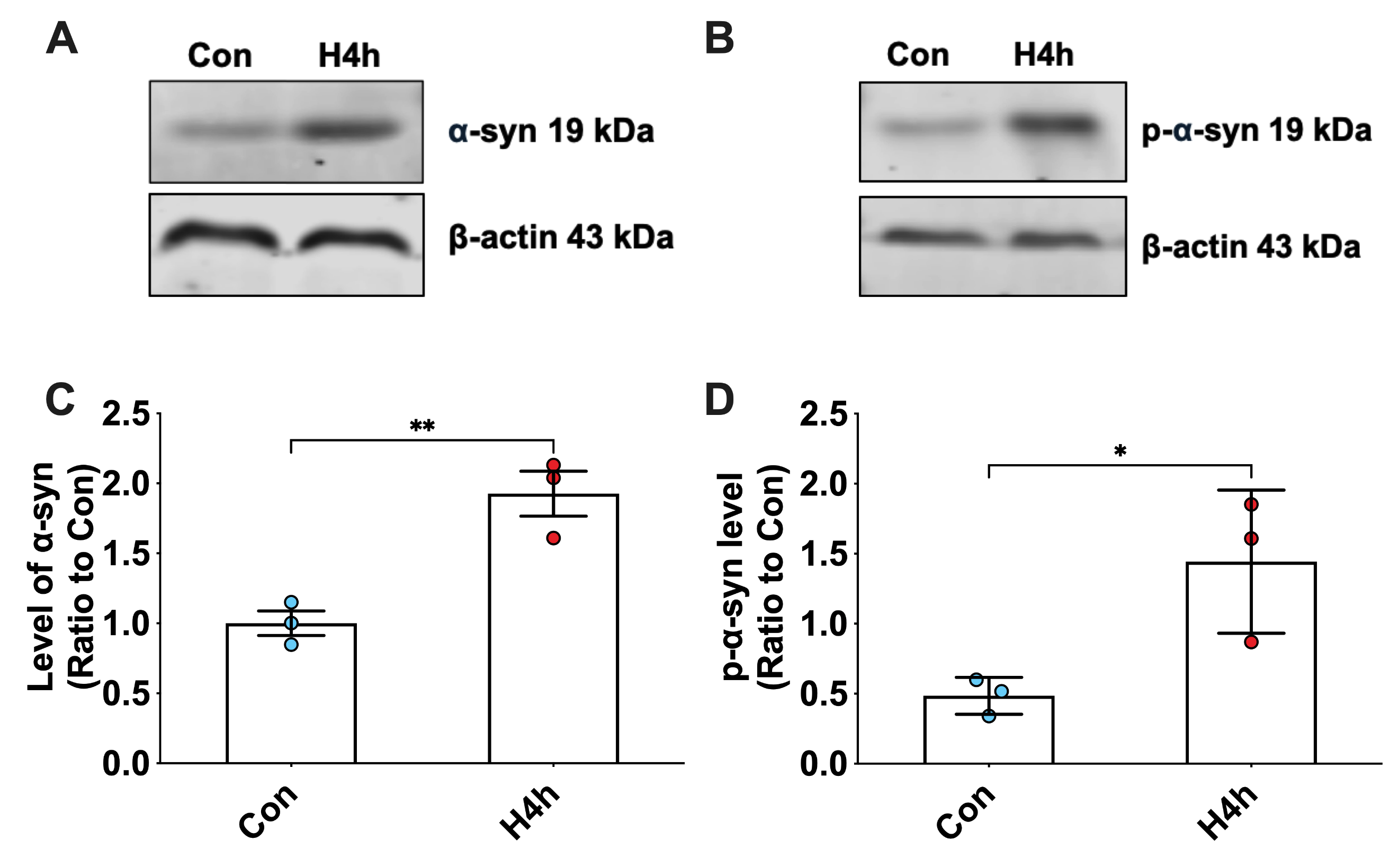


Original western blots


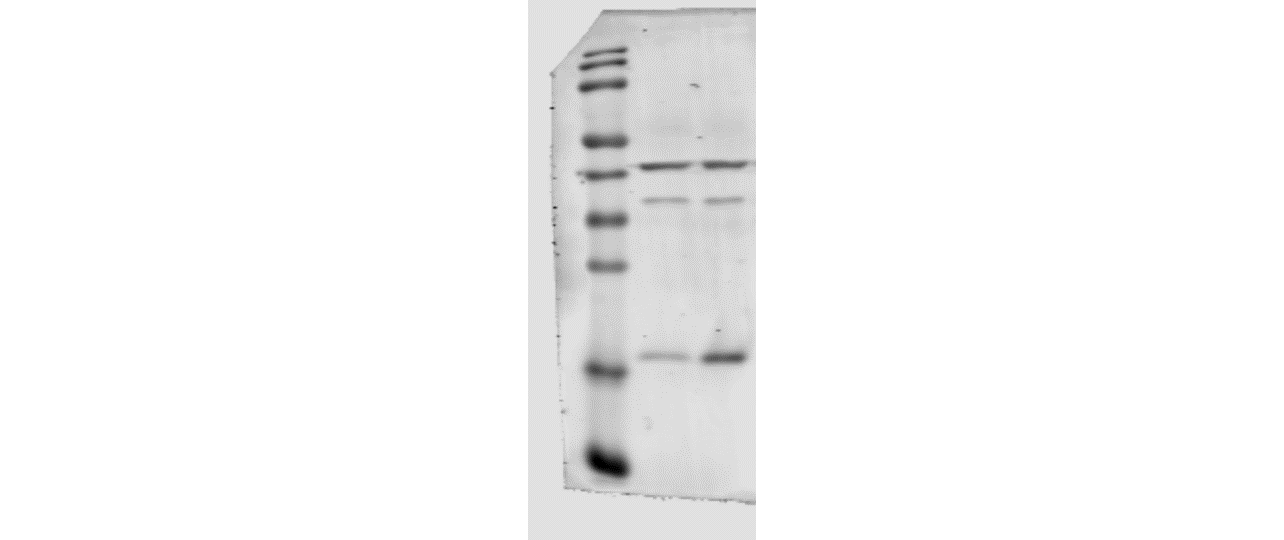


β-actin

p-α-syn

Figure S5A


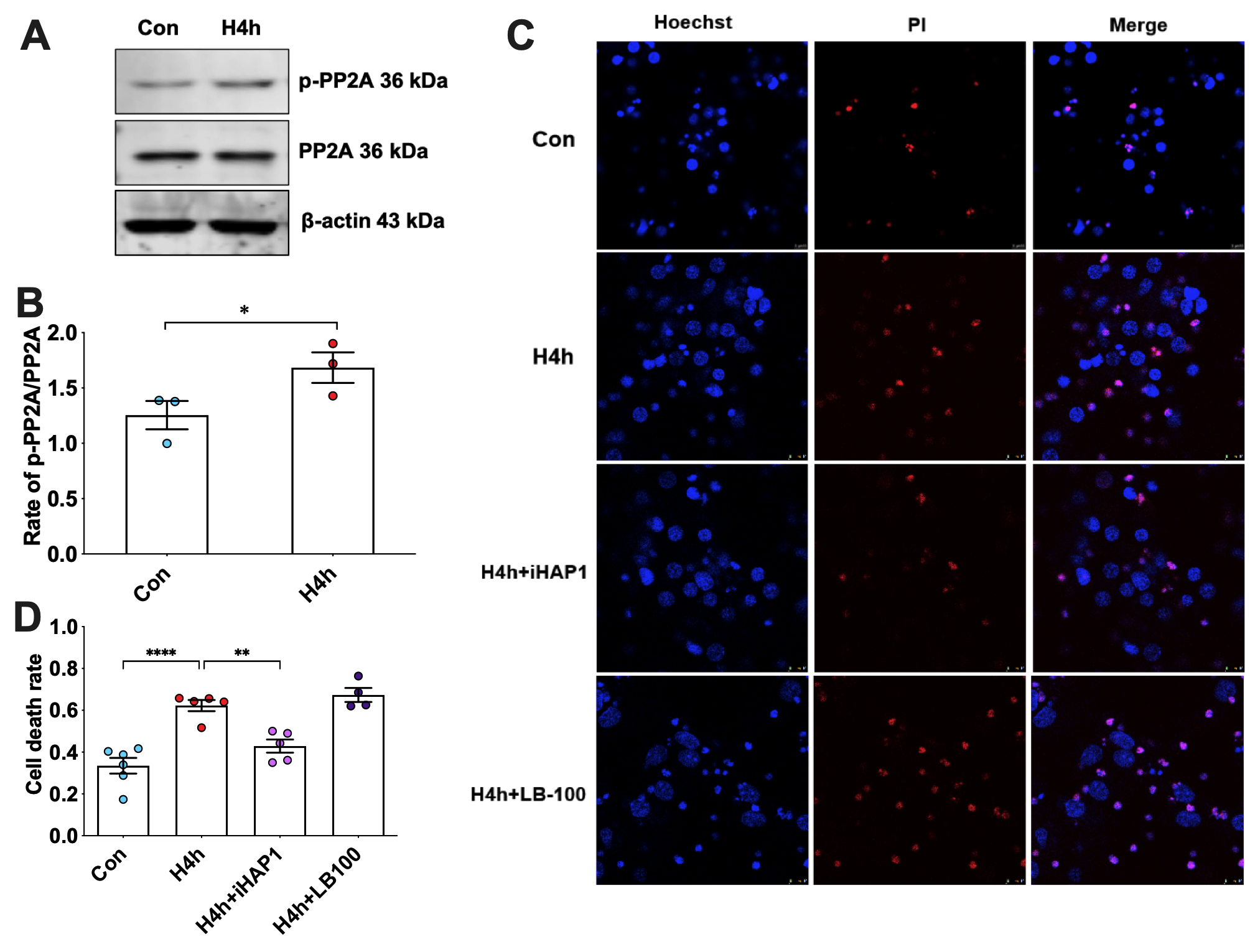


Original western blots

Con H4h


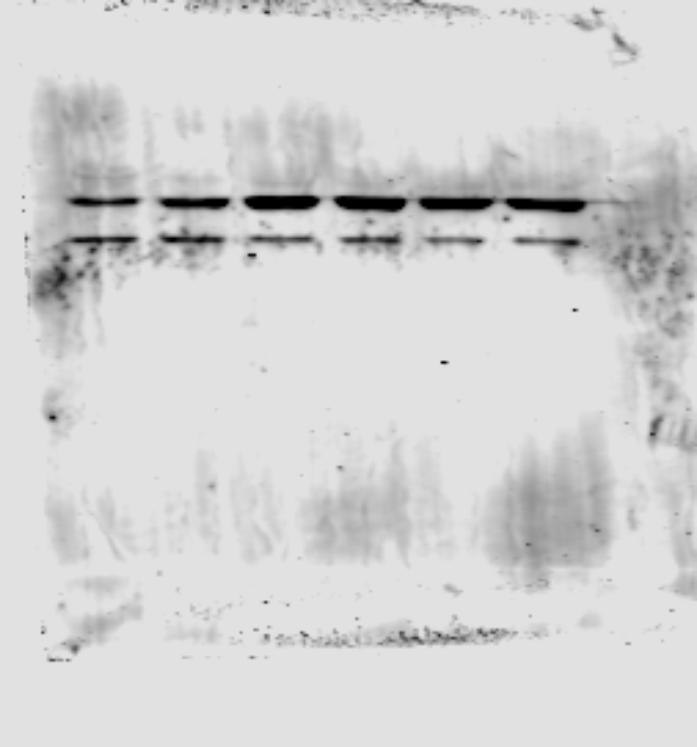


p-PP2A

β-actin

Con H4h


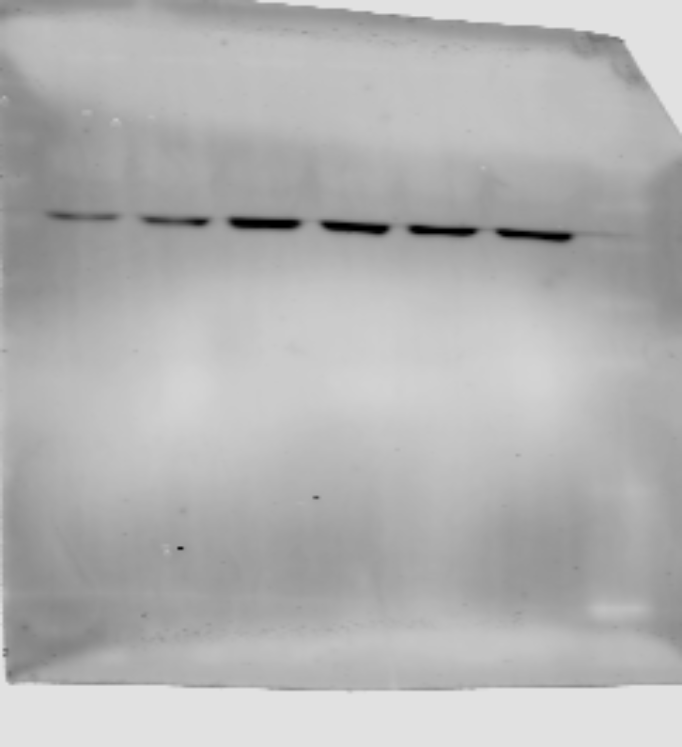


PP2A
